# Stem Cells in Cancer: From Mechanisms to Therapeutic Strategies

**DOI:** 10.3390/cells14070538

**Published:** 2025-04-03

**Authors:** Laurence Haddadin, Xueqin Sun

**Affiliations:** Cancer Genome and Epigenetics Program, NCI-Designated Cancer Center, Sanford Burnham Prebys Medical Discovery Institute, La Jolla, CA 92037, USA

**Keywords:** cancer, stem cells, signaling pathways, genetics, epigenetics, tumor microenvironment, epithelial–mesenchymal transition (EMT), extracellular matrix (ECM), tumor heterogeneity, metastasis

## Abstract

Stem cells have emerged as a pivotal area of research in the field of oncology, offering new insights into the mechanisms of cancer initiation, progression, and resistance to therapy. This review provides a comprehensive overview of the role of stem cells in cancer, focusing on cancer stem cells (CSCs), their characteristics, and their implications for cancer therapy. We discuss the origin and identification of CSCs, their role in tumorigenesis, metastasis, and drug resistance, and the potential therapeutic strategies targeting CSCs. Additionally, we explore the use of normal stem cells in cancer therapy, focusing on their role in tissue regeneration and their use as delivery vehicles for anticancer agents. Finally, we highlight the challenges and future directions in stem cell research in cancer.

## 1. Introduction

Cancer remains one of the leading causes of death worldwide, despite significant advances in diagnosis and treatment. Traditional cancer therapies, such as chemotherapy, radiation, and surgery, have improved patient outcomes, but they often fail to eradicate the disease completely, leading to recurrence and metastasis. The discovery of CSCs has provided a new paradigm for understanding cancer biology and has opened new avenues for therapeutic intervention [1,2,3,4]. By targeting CSCs, researchers aim to address the root causes of tumor resistance and relapse, paving the way for more effective cancer treatments. CSCs are a distinct subpopulation of tumor cells with stem cell-like properties, including self-renewal, differentiation, and the ability to evade cellular senescence, enabling them to initiate and sustain tumor growth [1,2,3,4,5,6,7]. CSCs are believed to be responsible for tumor initiation, progression, metastasis, and therapy resistance, making them a critical target for cancer treatment. This review provides an in-depth analysis of CSC biology, their roles in tumorigenesis and drug resistance, signaling pathways governing their behaviors, and potential therapeutic strategies aimed at eradicating CSCs during cancer treatment. Additionally, we will explore the use of normal stem cells in cancer therapy, including their role in tissue regeneration and their use as delivery vehicles for anticancer agents. Finally, we will highlight the challenges and future directions in CSC research.

## 2. Cancer Stem Cells (CSCs)

### 2.1. Origin of CSCs

**Figure 1:**
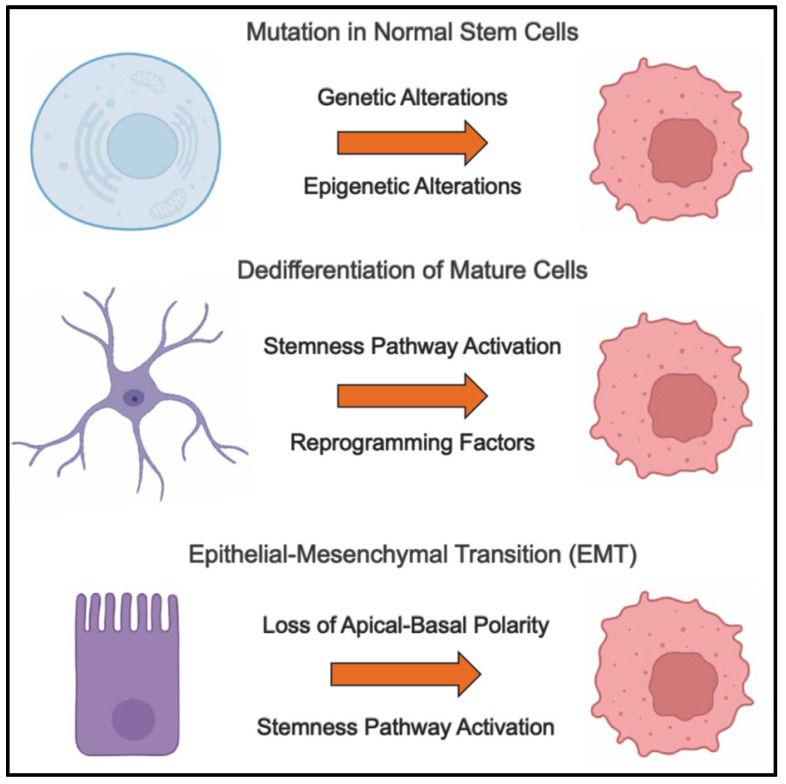
The origins of CSCs. Genetic and epigenetic alterations can accumulate in normal stem cells, driving them toward a tumorigenic phenotype. In differentiated cells, such as astrocytes, the activation of stemness and reprogramming factors may lead to dedifferentiation into cancer stem cells. Additionally, epithelial cells may lose their polarity and, alongside increased stemness, transform into CSCs that facilitate metastasis.

The origins of CSCs remain an area of active research, with several hypotheses proposed to explain their existence. The exact origin of CSCs may vary depending on the type of cancer and the specific genetic and epigenetic alterations involved. For example, in some cancers, such as leukemia, CSCs are thought to originate from hematopoietic stem or progenitor cells (HSPCs), while in solid tumors, CSCs may arise from tissue-specific stem cells or differentiated cells that have acquired stem cell-like properties [5,6,7] (Figure 1).

#### 2.1.1. Mutation in Normal Stem Cells

Cancer often arises from the accumulation of genetic and epigenetic alterations in normal cells, leading to uncontrolled growth and tumor formation. In many cases, normal stem cells, due to their inherent ability to self-renew and differentiate, serve as the cell of origin for cancers [5,6,7]. Their prolonged lifespan and frequent cell divisions make them particularly susceptible to oncogenic transformation. Normal stem cells are defined by two fundamental characteristics: self-renewal, the ability to generate identical daughter cells while maintaining an undifferentiated state, and multipotency, the ability to differentiate into multiple cell types, contributing to tissue homeostasis and repair. While these features are essential for tissue maintenance, they also increase the risk of accumulating mutations that can drive tumorigenesis. One hypothesis suggests that CSCs arise from normal stem cells or progenitor cells that acquire genetic and epigenetic alterations, leading to their transformation into cancer-initiating cells (CICs). As mutations accumulate in these cells, their regulatory mechanisms are disrupted, allowing them to initiate and sustain tumor growth.

The transformation of normal stem cells into CICs or CSCs typically involves a combination of genetic mutations and epigenetic alterations that disrupt normal cellular processes, leading to uncontrolled proliferation, survival, and stemness. Genetic mutations can occur in both oncogenes and tumor suppressor genes. Several key oncogenic mutations have been identified. RAS mutations (KRAS, NRAS, and HRAS) hyperactivate signaling pathways such as MAPK/ERK, promoting uncontrolled proliferation [8,9,10,11]. PIK3CA mutations lead to the constitutive activation of PI3K-AKT-mTOR, enhancing survival and metabolism [12,13,14,15]. MYC amplification drives metabolic reprogramming and increased stemness, contributing to aggressive tumor growth [16,17,18]. Except for oncogenic mutations, tumor suppressor alterations also play a crucial role in transforming normal stem cells into CSCs. For instance, p53 mutations or epigenetic silencing disrupt apoptosis and cell cycle control, allowing DNA-damaged stem cells to survive and proliferate [19,20,21,22,23,24,25,26]. RB1 loss promotes uncontrolled cell cycle progression [27,28,29,30], while PTEN deletions result in the hyperactivation of AKT signaling, enhancing survival and proliferation [31,32,33,34].

In addition to genetic mutations, epigenetic changes also play a crucial role in converting normal stem cells into CICs or CSCs. For example, hypermethylation of the CpG islands of tumor suppressor promoters (e.g., *CDKN2A* and *CHD5*) silences their expression, disrupting regulatory pathways that control cell growth and differentiation [35,36,37,38]. Moreover, aberrant histone modifications, such as the loss of H3K27me3 (often due to EZH2 mutations), lead to the activation of oncogenic genes and pathways [39,40,41]. Furthermore, dysregulation of chromatin remodelers (e.g., CHD5, BRG1, ARID1A, and BRD8) contributes to the loss of differentiation potential and enhanced stemness, promoting CSCs [26,42,43,44,45]. In leukemia, mutations in HSPCs—such as MLL fusions and FLT3-ITD—drive leukemic transformation [46,47,48,49]. Moreover, epigenetic mutations in DNMT3A and TET2 promote self-renewal and block differentiation, leading to the clonal expansion of leukemic stem cells (LSCs) [50,51].

#### 2.1.2. Dedifferentiation of Progenitor and Somatic Cells

While normal stem cells are a common source of CICs, recent evidence suggests that differentiated cells can also acquire stem cell-like properties through dedifferentiation or reprogramming, contributing to tumorigenesis. Dedifferentiation refers to the reversion of mature, lineage-committed cells to a less differentiated, stem-like state, often driven by oncogenic signals, stress conditions, or epigenetic reprogramming. This process allows non-stem cells to regain self-renewal capacity and plasticity, enabling them to initiate and sustain cancer growth.

The dedifferentiation and reprogramming of differentiated cells into cancer stem-like cells are driven by multiple molecular mechanisms. The activation of stemness pathways plays a key role, with Wnt/β-catenin signaling enhancing self-renewal and dedifferentiation [52,53,54], Notch signaling maintaining an undifferentiated state and tumor plasticity [55,56,57], and Hippo/YAP signaling promoting cell plasticity [58,59,60,61]. Epigenetic reprogramming also contributes, as EZH2 mutations or PRC2 repression leads to a stem-like chromatin state [62,63,64,65], while DNA methylation changes silence differentiation genes and activates stemness genes [35,37,66,67]. Transcriptional reprogramming through OCT4, SOX2, NANOG, and KLF4 (Yamanaka factors) induces pluripotency in cancer cells [68,69,70], and MYC overexpression promotes dedifferentiation by rewiring metabolic and transcriptional programs [16,18,71,72]. Metabolic shifts further sustain these cells, with a switch from oxidative phosphorylation to glycolysis (Warburg effect), supporting rapid proliferation, and enhanced glutamine metabolism and lipid biosynthesis, ensuring survival. Dedifferentiation has been observed in various contexts. For instance, astrocytes and oligodendrocytes can dedifferentiate into glioma stem cells (GSCs) through p53 loss, NF1 mutations, and EGFR activation, with hypoxia and inflammation further promoting plasticity [73,74,75,76]. Melanocytes, typically terminally differentiated, can revert to neural crest-like progenitors under stress [77,78]. The epithelial-to-mesenchymal transition (EMT), driven by ZEB1, TWIST, and SNAIL, represses epithelial markers (E-cadherin) and enhances stem-like properties, increasing metastasis, drug resistance, and tumor recurrence [79,80]. Differentiated intestinal enterocytes can become tumor-initiating cells in response to APC loss [81], Wnt activation [82], and inflammatory signals [83], while acinar cells in the pancreas dedifferentiate into ductal-like progenitors under KRAS activation, a key step in pancreatic ductal adenocarcinoma (PDAC) formation [84].

#### 2.1.3. Epithelial–Mesenchymal Transition (EMT)

Epithelial cells can undergo EMT, a process in which they lose polarity and adhesion properties [85,86]. This EMT transformation enhances their ability to move through the extracellular matrix (ECM) and invade surrounding tissues, contributing to cancer stemness, tumor progression, and metastasis. EMT is a key driver of cancer aggressiveness, enhancing therapy resistance and promoting metastatic seeding [87]. Epithelial cells are typically polarized, tightly adherent, and organized within tissue structures. During EMT, epithelial cells downregulate E-cadherin, a hallmark of epithelial integrity, while upregulating N-cadherin and vimentin, facilitating mesenchymal behavior [86]. They also lose apical–basal polarity through disruptions in the Par, Scrib, and Crumbs complexes, along with the loss of tight junction proteins (ZO-1 and Claudins) and cytoskeletal remodeling [86,88,89]. EMT enhances motility and invasion by increasing ECM degradation via MMPs and upregulating integrins for migration through the basement membrane. Furthermore, EMT induces CSC-like properties, as EMT transcription factors (ZEB1, SNAIL, and TWIST) activate pluripotency regulators (SOX2, OCT4, and NANOG) while sustaining stemness through the Wnt/β-catenin, Notch, and Hedgehog pathways [79,90,91,92,93,94]. EMT-associated CSCs exhibit enhanced drug resistance via ABCB1 and ABCG2 transporters [95], apoptosis evasion [96], and immune resistance [97], allowing them to enter a quiescent state, evade treatment, and later reactivate as metastatic lesions [98]. Basal-like triple-negative breast cancer (TNBC) shows strong EMT features, with ZEB1 repressing differentiation genes and activating stemness programs [99]. In colorectal cancer, Wnt/β-catenin signaling induces EMT and CSC-like traits, while APC loss sustains stemness and tumor initiation [93,100]. In lung cancer, TGF-β and hypoxia drive EMT in non-small cell lung cancer (NSCLC), leading to chemoresistance and immune evasion [101,102,103]. In PDAC, KRAS-driven EMT correlates with poor prognosis and metastasis [104,105]. Additionally, CSCs may arise through cell fusion or the uptake of extracellular vesicles containing oncogenic material, further diversifying tumor cell plasticity [106,107,108].

### 2.2. Identification and Isolation of CSCs

The identification and isolation of CSCs have been challenging due to their heterogeneity and the lack of specific markers. However, several methods have been developed to identify and isolate CSCs based on their unique properties, including surface markers, functional assays, and gene expression profiles. CSCs often express specific surface markers that distinguish them from non-stem cancer cells. These markers vary across cancer types but are widely used for CSC enrichment using flow cytometry or magnetic-activated cell sorting (MACS). For instance, in hematological malignancies, CD34^+^/CD38^−^ identifies LSCs in acute myeloid leukemia (AML) [109,110]. CD133 (Prominin-1) is a common marker in glioblastoma, colon, prostate, gastric, and liver cancers [111,112]. ALDH1 (Aldehyde Dehydrogenase 1) serves as a metabolic marker for CSCs in breast, lung, glioblastoma, and ovarian cancers [113,114,115]. EpCAM (Epithelial Cell Adhesion Molecule) is a marker for colorectal, breast, hepatocellular carcinoma, and pancreatic CSCs, with LGR5, CD133, and CD44 specifically identifying colorectal CSCs (CRCSCs) [116,117,118]. GSCs are characterized by CD133, SOX2, and Nestin [113,119], whereas CD44^+^/CD24^−^ is used for breast CSCs [120]. While these markers are useful, their heterogeneous and context-dependent expression necessitates complementary approaches. Functionally, CSCs exhibit the ability to grow as non-adherent spheres in serum-free conditions, growth factor-enriched conditions, mimicking their self-renewal ability in vivo [121]. Moreover, CSCs can be identified by their high drug efflux capacity, which is detectable using Hoechst 33342 dye exclusion [122], and their ALDH activity, measured by the ALDEFLUOR assay [123]. Furthermore, CSCs exhibit high resistance to chemotherapy and radiation, allowing their enrichment post-treatment. Additionally, gene expression profiling has been instrumental in CSC identification, as CSCs often express stem cell maintenance genes such as OCT4, SOX2, and NANOG, as well as drug resistance genes like ABC transporters [68,70,95]. These gene expression signatures can be analyzed using RNA sequencing to isolate CSCs. Advancements in single-cell and multi-omics technologies have further revolutionized CSC identification. Single-cell RNA sequencing (scRNA-seq) enables the characterization of CSC heterogeneity, epigenetic profiling reveals CSC-specific chromatin modifications, and lineage tracing allows for the tracking of CSC fate in tumors. The gold standard for CSC identification remains the in vivo tumor initiation assay, where xenograft models and limiting dilution assays in immunocompromised mice assess tumor-forming potential, with CSCs requiring significantly fewer cells to initiate tumors compared to bulk cancer cells.

### 2.3. Signaling Pathways in CSCs

**Figure 2:**
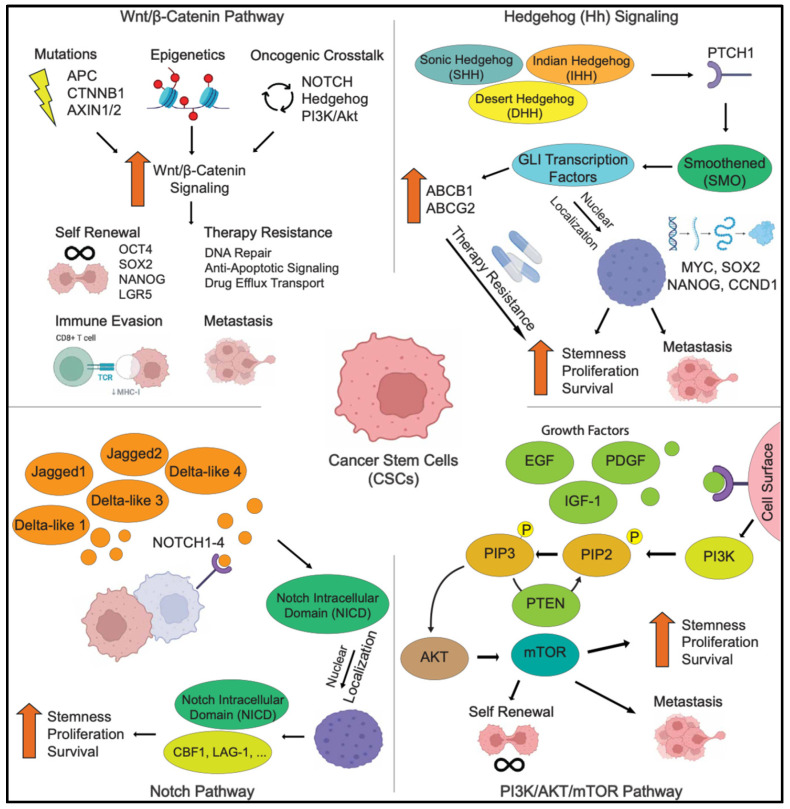
The signaling pathways of CSCs. A schematic illustrating key signaling pathways associated with CSCs, including the Wnt/β-Catenin, Hedgehog (Hh), Notch, and PI3K/AKT/mTOR pathways.

CSCs are regulated by various intracellular and extracellular signaling pathways that control their self-renewal, differentiation, survival, and therapy resistance. Many of these pathways are shared with normal stem cells but become dysregulated in CSCs, promoting tumorigenesis and resistance to treatment (Figure 2).

#### 2.3.1. The Wnt/β-Catenin Signaling Pathway

The Wnt/β-catenin signaling pathway plays a pivotal role in embryonic development, tissue homeostasis, and stem cell maintenance [53,54,124,125]. The pathway operates through two main branches: canonical (β-catenin-dependent) signaling, which regulates gene transcription, and non-canonical (β-catenin-independent) signaling, which influences cell polarity and migration. In CSCs, hyperactivation of Wnt/β-catenin signaling occurs due to genetic mutations (e.g., APC, CTNNB1, or AXIN1/2), autocrine Wnt ligand production and paracrine signaling from the tumor microenvironment (TME). Epigenetic modifications, and crosstalk with other oncogenic pathways such as Notch, Hedgehog, and PI3K/Akt, also enhance Wnt signaling [126,127,128]. The aberrant activation of Wnt/β-catenin signaling is a hallmark of CSCs, promoting CSC self-renewal, tumor initiation, metastasis, and therapy resistance [93,94,129]. The aberrant activation of this pathway has been implicated in multiple cancers, including glioblastoma, colorectal, breast, and liver cancers, making it an attractive therapeutic target [94].

Wnt/β-catenin signaling plays a key role in CSC maintenance by promoting self-renewal through the upregulation of stemness-associated genes such as OCT4, SOX2, NANOG, and LGR5 [125,129]. Additionally, it contributes to therapy resistance by enhancing DNA repair mechanisms [130,131], activating anti-apoptotic signaling [132], and upregulating drug efflux transporters like ABCB1, which reduce chemotherapy efficacy [133,134]. This pathway also facilitates tumor metastasis by inducing EMT, a process that enables CSCs to acquire migratory and invasive properties [93]. Furthermore, Wnt signaling contributes to immune evasion by suppressing dendritic cell activation and preventing T cell infiltration, creating an immunosuppressive tumor microenvironment that allows CSCs to evade immune surveillance [135,136,137].

The impact of Wnt/β-catenin signaling varies across different CSC populations [94,125,129,138]. In glioblastoma, this pathway is crucial for GSC maintenance and therapy resistance, and its inhibition reduces tumor recurrence. In colorectal cancer, mutations in APC or β-catenin lead to constitutive Wnt activation, sustaining CSC populations and driving tumor initiation. Similarly, in breast cancer, particularly TNBC, high Wnt activity correlates with tumor aggressiveness and EMT induction, promoting metastasis and resistance to conventional treatments. Given its central role in CSC biology, targeting Wnt/β-catenin signaling represents a promising therapeutic strategy to eliminate CSCs and improve treatment outcomes across multiple cancer types [138].

#### 2.3.2. The Hedgehog Signaling Pathway

The Hedgehog (Hh) signaling pathway plays a crucial role in stem cell maintenance and is frequently dysregulated in CSCs, contributing to tumor growth, metastasis, and therapy resistance [94,127,139]. The pathway is primarily mediated by three ligands—Sonic Hedgehog (SHH), Indian Hedgehog (IHH), and Desert Hedgehog (DHH)—which signal through the Patched-1 (PTCH1) receptor to regulate Smoothened (SMO) activity. Upon activation, SMO initiates a signaling cascade that leads to the nuclear translocation of GLI transcription factors, which drive the expression of genes involved in stemness, proliferation, and survival, including NANOG, SOX2, MYC, and CCND1. The aberrant activation of this pathway can result from PTCH1 mutations, SMO activation, or GLI amplification, sustains CSC populations, and enhances tumor progression in various cancers [139,140]. Consequently, the hyperactivation of this pathway leads to the upregulation of stemness-associated genes, such as GLI1 and GLI2 transcription factors, promoting CSC self-renewal, proliferation, and survival. Furthermore, GLI1 activation upregulates ABC transporters, such as ABCG2 and ABCB1, which promote drug efflux and chemoresistance [141,142,143]. Moreover, it contributes to therapy resistance by enhancing DNA repair mechanisms and anti-apoptotic signaling [144,145]. Hedgehog signaling also drives EMT, increasing CSC invasiveness by upregulating transcription factors like Snail, Twist, and Zeb1, which suppress E-cadherin and enhance migration [146,147]. Additionally, Hedgehog signaling contributes to immune evasion by creating an immunosuppressive tumor microenvironment, reducing T cell infiltration and inhibiting immune cell activation, thereby enabling CSC survival and expansion [148,149].

The Hedgehog pathway plays a distinct role in maintaining CSCs across multiple cancer types [94,139]. In glioblastoma, Hedgehog signaling is highly active in GSCs, promoting tumor growth and resistance to temozolomide, while its inhibition reduces glioblastoma recurrence. In pancreatic cancer, Hedgehog signaling is critical for sustaining CSC populations and promoting aggressive tumor progression and chemoresistance, making Hh inhibitors a potential therapeutic approach to sensitize tumors to chemotherapy. Similarly, in TNBC, Hedgehog signaling is associated with enhanced CSC survival and tumor initiation capacity, with pathway inhibition reducing BCSC self-renewal. Given its central role in CSC biology, targeting key components of the Hedgehog pathway, such as SMO and GLI, represents a promising therapeutic strategy for eliminating CSCs and improving treatment outcomes.

#### 2.3.3. The Notch Signaling Pathway

The Notch signaling pathway is a highly conserved cell–cell communication system that plays a crucial role in stem cell maintenance, differentiation, and tissue homeostasis. In cancer, aberrant Notch signaling is frequently implicated in the regulation of CSCs, contributing to their self-renewal, survival, and resistance to therapy [55,56,57,127]. The pathway is activated through the interaction of Notch receptors (Notch1–4) with ligands (Jagged1, Jagged2, Delta-like 1, Delta-like 3, and Delta-like 4) on adjacent cells, requiring direct cell–cell contact. Upon ligand binding, the Notch receptor undergoes cleavage by the γ-secretase complex, releasing the Notch intracellular domain (NICD), which translocates to the nucleus. There, NICD binds to CSL (CBF1/Suppressor of Hairless/LAG-1) and Mastermind-like proteins to activate the expression of target genes such as HES, HEY, MYC, and NANOG, all of which are involved in maintaining stemness and promoting tumorigenesis.

Dysregulated Notch signaling enhances the self-renewal and tumor-initiating capacity of CSCs by upregulating key stemness factors like NANOG, SOX2, and OCT4. The activation of Notch1 and Notch2 has been shown to sustain CSC populations in several cancers, including glioblastoma, breast cancer, pancreatic cancer, and colorectal cancer. Additionally, Notch signaling contributes to therapy resistance by promoting chemoresistance and radioresistance [55,56,127]. This is achieved through the induction of ABC transporters, such as ABCG2, which promote drug efflux and reduce the efficacy of chemotherapy. Furthermore, Notch activation activates anti-apoptotic pathways, helping CSCs evade therapy-induced cell death [55]. The pathway also drives EMT, enhancing CSC invasion and metastasis, and promotes tumor angiogenesis through Jagged1-mediated signaling, which creates a supportive microenvironment for CSCs. Additionally, Notch signaling modulates tumor immune evasion by creating an immunosuppressive microenvironment that supports CSC survival. It promotes T cell exhaustion and macrophage polarization into a pro-tumorigenic, M2-like phenotype, which suppresses anti-tumor immunity and aids CSC persistence. By influencing immune components, Notch signaling helps CSCs resist immune surveillance, complicating therapeutic strategies [56,150].

Notch signaling is particularly critical in maintaining CSCs in specific cancers [55,56]. In glioblastoma, Notch1 and Notch2 are highly active in glioblastoma stem cells, where they sustain self-renewal and contribute to therapy resistance. The inhibition of Notch signaling reduces glioblastoma recurrence and enhances the sensitivity of GSCs to radiation. In TNBC, Jagged1-Notch1 signaling is associated with increased aggressiveness and EMT, promoting CSC survival and metastasis. Notch signaling also plays a central role in pancreatic cancer, where it supports CSC survival and chemoresistance. Notch inhibition has been shown to sensitize pancreatic cancer cells to chemotherapy, such as gemcitabine, making Notch a promising therapeutic target for overcoming CSC-mediated resistance and improving treatment outcomes in various cancers.

#### 2.3.4. The PI3K/AKT/mTOR Signaling Pathway

The PI3K/AKT/mTOR pathway is a key regulator of cell growth, metabolism, survival, and proliferation, playing an essential role in CSCs [151,152,153]. It maintains CSC self-renewal, contributes to therapy resistance, and enhances tumorigenicity. The aberrant activation of this pathway is implicated in the aggressiveness and persistence of CSCs, making it a potential therapeutic target in cancers such as glioblastoma, breast, pancreatic, and colorectal cancers. The signaling cascade begins when growth factors like EGF, IGF-1, and PDGF bind to receptors on the cell surface, triggering the activation of PI3K [151]. PI3K phosphorylates phosphatidylinositol-4,5-bisphosphate (PIP2) into PIP3, which then recruits AKT to the membrane. AKT activation regulates various downstream effectors involved in cell survival, metabolism, and proliferation. One of the key downstream targets of AKT is mTOR (mechanistic target of Rapamycin), a critical regulator of protein synthesis, autophagy, and stemness in CSCs. The tumor suppressor PTEN negatively regulates PI3K/AKT signaling by converting PIP3 back to PIP2, preventing AKT activation. The loss of PTEN function in CSCs leads to sustained PI3K/AKT/mTOR signaling, further promoting tumor growth and therapy resistance.

In CSCs, the PI3K/AKT/mTOR pathway supports self-renewal and tumor-initiating capacity by upregulating stemness-related genes such as OCT4, SOX2, NANOG, and MYC [154,155,156]. It also promotes therapy resistance by enhancing DNA repair mechanisms, increasing drug efflux through transporters like ABCG2, and protecting CSCs from cell death through mTOR activation. Additionally, AKT signaling supports CSC metabolism by promoting glycolysis, lipid biosynthesis, and mitochondrial function, providing energy for CSCs. mTORC1 activation further enhances protein synthesis and cellular biomass, driving tumor growth. In the hypoxic tumor microenvironment, PI3K/AKT signaling cooperates with HIF-1α to support CSC survival. Moreover, this pathway also facilitates CSC invasion and metastasis by phosphorylating transcription factors like TWIST and SNAIL, which are crucial for EMT. Targeting the PI3K/AKT/mTOR pathway has shown potential in reducing CSC maintenance, improving therapeutic responses, and limiting tumor progression across several cancer types [151,154]. In glioblastoma, PI3K/AKT/mTOR is frequently activated due to EGFR mutations and PTEN loss, and targeting mTOR signaling reduces GSC self-renewal and tumor growth. In TNBC, PI3K/AKT signaling enhances CSC expansion and chemoresistance, and PI3K inhibitors combined with chemotherapy improve CSC eradication. Moreover, in pancreatic cancer, PI3K/AKT/mTOR activation sustains CSCs and drives gemcitabine resistance.

### 2.4. Cancer Stem Cell Niche

**Figure 3:**
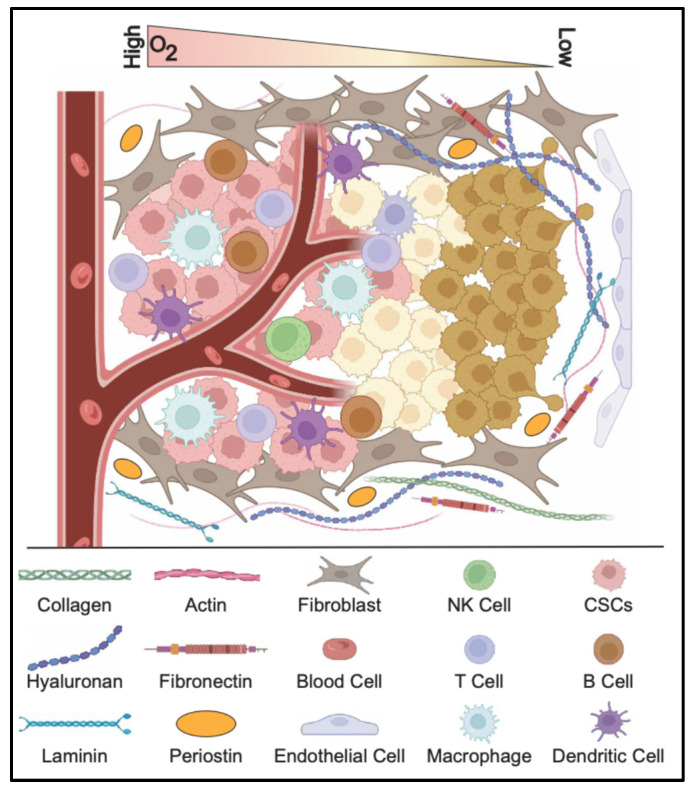
The CSC niche. The tumor microenvironment sustains CSC survival and function while inhibiting immune cell activity and promoting hypoxia.

The CSC niche is a specialized microenvironment that supports CSC survival, self-renewal, and resistance to therapy. It consists of stromal cells, ECM proteins, signaling molecules, and immune cells that interact with CSCs to regulate their behavior. The niche is often characterized by hypoxia, immune modulation, and stromal components that protect CSCs from conventional treatments, contributing to tumor recurrence (Figure 3).

#### 2.4.1. Hypoxia and Acidity

Hypoxia, a hallmark of the tumor microenvironment, plays a crucial role in shaping the CSC niche by promoting stemness and enhancing tumor aggressiveness [102,157,158,159]. Hypoxic conditions stabilize hypoxia-inducible factors (HIF-1α and HIF-2α), which activate the transcription of stemness-associated genes such as OCT4, SOX2, NANOG, and c-MYC. These factors reinforce CSC self-renewal and prevent differentiation, maintaining the CSC pool within the tumor. Additionally, HIF signaling promotes EMT, increasing CSC plasticity and invasive potential. Hypoxia-driven metabolic reprogramming further enhances CSC survival and adaptation [102]. Under low-oxygen conditions, CSCs shift towards glycolysis, increasing glucose uptake and lactate production to sustain energy demands. This metabolic adaptation supports CSC proliferation and creates an immunosuppressive microenvironment that inhibits T cell and NK cell activity. Moreover, hypoxia induces the secretion of vascular endothelial growth factor (VEGF), promoting angiogenesis and sustaining the tumor’s oxygen supply. The newly formed blood vessels are often leaky and disorganized, perpetuating a chronic hypoxic state that reinforces CSC stemness and therapy resistance.

In addition to metabolic and transcriptional changes, hypoxia contributes to therapy resistance in CSCs by upregulating drug efflux transporters such as ABCG2, which actively remove chemotherapeutic agents from the cell [158,159,160]. Hypoxia also enhances resistance to radiation therapy by reducing reactive oxygen species (ROS) production, thereby preventing radiation-induced DNA damage. Furthermore, hypoxic CSCs secrete extracellular vesicles containing pro-survival and immunosuppressive factors, which modulate the tumor microenvironment and protect CSCs from immune attack. Targeting hypoxia-driven pathways, including HIF inhibitors and metabolic modulators, presents a promising strategy to disrupt the CSC niche and improve cancer treatment outcomes.

Similarly, an acidic extracellular pH (~6.5–6.8) in the tumor microenvironment serves as a harsh selective pressure that is detrimental to normal cells yet supports the maintenance and survival of CSCs. In gliomas, chronic acidosis (pH 6.5) increases the expression of core stemness genes like OCT4 and NANOG, and stabilizes HIF-2α, driving a more stem-like, angiogenic phenotype [161,162]. Notably, pancreatic tumor cells adapted to a low pH develop heightened Wnt/β-catenin signaling, increased ALDH activity, greater spheroid formation, and in vivo metastatic capacity, indicating that acidic stress promotes CSC traits [163]. Acidosis triggers several adaptive mechanisms that allow CSCs to thrive and resist therapy. Acid-exposed CSCs upregulate proton transporters (Na^+^/H^+^ exchangers and Vacuolar H^+^-ATPase) and carbonic anhydrases to maintain a near-neutral intracellular pH, enhance glycolytic and autophagic pathways for survival, and often enter a slow-cycling state that evades treatment [164,165,166]. Additionally, an acidic milieu impairs the efficacy of certain chemotherapeutics (via drug protonation and “ion trapping”) selecting for CSCs with elevated stress chaperones (GRP78) and drug efflux pumps (ABCG2, P-gp), developing a multidrug-resistant phenotype [167,168,169]. These adaptations allow CSCs to thrive under acidic conditions and contribute to therapy resistance.

#### 2.4.2. Immune Evasion

CSCs employ multiple strategies to evade immune detection and persist under immune surveillance within the niche [170,171,172]. A key mechanism involves the modulation of immune checkpoint molecules such as PD-L1, which enables CSCs to escape T cell-mediated immunity. By upregulating PD-L1, CSCs interact with PD-1 on T cells, inducing immune exhaustion and suppressing effective anti-tumor responses. Additionally, CSCs overexpress other immune checkpoint molecules, including CD47 and Galectin-9, which further inhibit T cell activation. CD47 interacts with SIRPα on macrophages, delivering a “don’t eat me” signal that prevents phagocytosis. To avoid immune recognition, CSCs also downregulate tumor antigens and antigen-presenting molecules such as MHC-I and MHC-II, reducing their visibility to cytotoxic T lymphocytes (CTLs) and facilitating immune escape.

CSCs actively manipulate the tumor microenvironment to suppress immune responses [173,174,175]. They recruit regulatory T cells (Tregs) by secreting immunosuppressive cytokines such as TGF-β, IL-10, and indoleamine 2,3-dioxygenase (IDO), which inhibit effector T cell functions. Additionally, CSCs foster an immunosuppressive microenvironment by secreting factors like VEGF, IL-6, and IL-10, which suppress CTL and NK cell activity. Metabolic alterations, including the increased expression of IDO and Arginase-1 (ARG1), further inhibit T cell function by depleting essential amino acids such as tryptophan and arginine. CSCs also promote myeloid-derived suppressor cells (MDSCs) and tumor-associated macrophages (TAMs) with an M2-like phenotype, which further dampen immune responses. To evade NK cell-mediated killing, CSCs downregulate NKG2D ligands and overexpress HLA-G, impairing NK cell recognition. Additionally, they upregulate FasL and TRAIL decoy receptors, rendering them resistant to NK cell-induced apoptosis.

Hypoxia within the tumor microenvironment further enhances CSC immune evasion [102,157,176]. Hypoxia-induced HIF-1α signaling upregulates PD-L1 expression and suppresses antigen presentation, reinforcing immune resistance. CSCs also shift their metabolism towards aerobic glycolysis, increasing lactate production, which contributes to an immunosuppressive environment that inhibits T cell function. Furthermore, CSCs interact with cancer-associated fibroblasts (CAFs) to maintain immune suppression and secrete immunosuppressive factors via extracellular vesicles (exosomes), further modulating immune cell activity. Prolonged exposure to CSC-derived factors leads to the exhaustion of CD8+ T cells, marked by the elevated expression of PD-1, TIM-3, and LAG-3 [173,177,178]. Exhausted T cells lose their ability to produce IFN-γ, TNF-α, and IL-2, ultimately diminishing their anti-CSC function. CSCs also reside near blood vessels, where endothelial-derived factors such as VEGF and Notch ligands support their stemness [179,180]. In line with this, disrupting this perivascular niche can impair GSC self-renewal and tumor growth. Collectively, these mechanisms allow CSCs to evade immune surveillance, drive tumor progression, and contribute to resistance against immunotherapies.

#### 2.4.3. Extracellular Matrix Interactions

The CSC niche is a specialized microenvironment composed of ECM components, stromal cells, and soluble factors that sustain CSC properties [181,182]. Within this niche, CSCs interact with fibroblasts, endothelial cells, and immune cells to maintain their stemness and drive tumor progression. The ECM plays a crucial role in regulating CSC self-renewal, therapy resistance, and metastatic potential by providing structural support and biochemical signals. Through interactions with integrins, growth factor signaling, and mechanotransduction pathways, the ECM influences CSC behavior, making it a critical target for disrupting the TME and limiting CSC-driven tumor growth.

Key ECM components, including collagens, fibronectin, laminins, hyaluronic acid (HA), and periostin, actively regulate CSC survival and function [183,184]. Collagen types I and IV promote CSC maintenance and therapy resistance through integrin-mediated activation of the PI3K/AKT and Wnt/β-catenin pathways [185,186]. Integrin-FAK signaling facilitates ECM adhesion, enhancing CSC invasion and resistance to treatment. Fibronectin interactions with integrins (α5β1 and αvβ3) enhance CSC adhesion, migration, and drug resistance, contributing to tumor relapse. Laminins, particularly in brain and breast cancers, sustain CSCs by activating Notch and integrin signaling, contributing to therapy resistance [187,188]. HA interacts with CD44, a key CSC marker, to drive self-renewal, drug resistance, and activation of oncogenic pathways such as YAP/TAZ, Wnt/β-catenin, and PI3K/AKT [189,190,191]. Periostin further supports CSC expansion in breast and colorectal cancers by modulating Wnt and integrin signaling, reinforcing the role of ECM in CSC maintenance [192,193,194]. Additionally, ECM stiffness and HA-CD44 interactions regulate YAP/TAZ activity, reinforcing CSC survival, metastasis, and drug resistance [183]. These intricate ECM-mediated mechanisms highlight the importance of targeting the CSC niche to develop more effective cancer therapies and limit tumor recurrence.

### 2.5. Role of CSCs in Tumorigenesis

CSCs play a critical role in tumorigenesis, as they are believed to be the cells responsible for initiating and sustaining tumor growth. CSCs have the ability to self-renew, allowing them to maintain a pool of undifferentiated cells within the tumor, and to differentiate into various cell types, contributing to tumor heterogeneity.

#### 2.5.1. Self-Renewal and Differentiation

The self-renewal and differentiation of CSCs are tightly regulated processes that sustain tumor growth and contribute to therapy resistance [5,6,195,196,197]. CSCs maintain their stem-like properties through key signaling pathways, including Wnt/β-catenin, Notch, Hedgehog, and PI3K/AKT, which regulate their ability to self-renew and resist differentiation cues. These pathways interact with the TME, where niche components such as fibroblasts, endothelial cells, and immune cells provide signals that support CSC maintenance. Additionally, hypoxia, a common feature of the CSC niche, further enhances self-renewal by stabilizing HIF-1α, which upregulates stemness-associated genes and promotes a quiescent state that protects CSCs from chemotherapy and immune attack.

Despite their ability to self-renew, CSCs can also differentiate into heterogeneous tumor cell populations, contributing to intra-tumoral diversity and tumor progression [198]. This differentiation is often regulated by microenvironmental cues, including growth factors, cytokines, and ECM interactions. However, unlike normal stem cells, CSC differentiation is often incomplete or reversible, allowing them to regain stem-like properties under stress conditions such as chemotherapy or radiation. This plasticity enables CSCs to adapt to changing microenvironments and contributes to tumor relapse. Factors such as TGF-β, IL-6, and interactions with mesenchymal stromal cells can promote an EMT-like state, further enhancing CSC invasiveness and metastatic potential [199,200]. Key regulatory factors, including SOX2, OCT4, NANOG, and EMT-associated proteins such as ZEB1, SNAIL, and TWIST, suppress differentiation while enhancing invasion and metastatic potential [5,196,201]. Moreover, epigenetic mechanisms such as DNA methylation, histone modifications (e.g., increased H3K27me3, often by EZH2), and non-coding RNAs (e.g., miR-34a) further regulate CSC fate by silencing differentiation genes and sustaining self-renewal pathways [202,203,204,205]. A deeper understanding of CSC plasticity and differentiation mechanisms is crucial for developing targeted therapies that prevent tumor recurrence and metastasis.

#### 2.5.2. Tumor Heterogeneity

Tumor heterogeneity is a defining feature of cancer, driven by genetic mutations, epigenetic modifications, and interactions with the tumor microenvironment. CSCs play a pivotal role in this diversity by generating various cancer cell subpopulations with distinct genetic, phenotypic, and functional properties [198,206,207,208]. CSCs contribute to tumor heterogeneity by undergoing dynamic epigenetic regulation, allowing them to switch between stem-like and differentiated states. Unlike normal stem cells, CSC differentiation is often incomplete or reversible, enabling them to adapt to changing microenvironments and therapeutic pressures. Hypoxia and inflammation further promote CSC heterogeneity by activating pathways such as HIF-1α and Notch, enhancing survival and plasticity [207]. Inflammation and immune evasion contribute to the survival of distinct CSC subpopulations [206]. The hierarchical organization of tumors resembles normal stem cell differentiation, with CSCs generating diverse progenitor and differentiated tumor cells. This intra-tumoral complexity poses significant challenges for cancer treatment, as different subpopulations may exhibit varying responses to therapy, leading to drug resistance and disease recurrence. Understanding the mechanisms underlying CSC-driven heterogeneity is crucial for developing targeted therapies that can effectively eradicate CSCs, prevent adaptation, and improve treatment outcomes.

Tumor heterogeneity significantly complicates the development of CSC-targeted therapies, as different CSC subpopulations may rely on distinct signaling pathways or exhibit varying degrees of drug sensitivity. To address this, combination therapies targeting multiple CSC-related pathways (e.g., Wnt, Notch, Hedgehog, and PI3K/Akt) are being investigated to ensure a broader coverage of heterogeneous CSC populations. Additionally, epigenetic modulators, such as HDAC and DNMT inhibitors, can limit CSC plasticity and reduce the emergence of resistant subpopulations. Immunotherapeutic strategies—including CAR-T cells and immune checkpoint inhibitors—also offer promise by enabling the immune system to recognize and eliminate diverse CSC subsets. Personalized medicine approaches, leveraging biomarkers to stratify patients based on CSC characteristics, may further enhance the precision and efficacy of these therapies in overcoming heterogeneity and preventing tumor recurrence.

### 2.6. Role of CSCs in Metastasis

Metastasis is the spread of cancer cells from the primary tumor to distant organs, forming secondary tumors. CSCs are key drivers of metastasis, as they can invade tissues, enter the bloodstream, and colonize distant sites. CSCs contribute to metastasis through their self-renewal capacity, plasticity, resistance to apoptosis, and immune evasion, allowing them to survive during dissemination and establish secondary tumors.

#### 2.6.1. Epithelial–Mesenchymal Transition (EMT)

CSCs often undergo EMT, which enables them to invade surrounding tissues and enter the bloodstream, facilitating metastasis [85,209,210]. EMT is tightly regulated by various signaling pathways, including TGF-β, Wnt, and Notch. In CSCs, these pathways are frequently dysregulated, contributing to their enhanced migratory ability and metastatic potential. Once in circulation, CSCs can colonize specific microenvironments known as “pre-metastatic niches”, where they receive signals that support their survival and proliferation. In the bone marrow, CSCs interact with osteoblasts and bone stromal cells, a process commonly seen in breast and prostate cancer metastasis. In brain metastases, CSCs take advantage of the neurovascular niche, where astrocytes provide protection and support [211,212,213,214]. Similarly, in the liver and lungs, CSCs engage with fibroblasts, immune cells, and extracellular matrix components to establish secondary tumors and promote metastatic growth. In particular, the CAFs that interact with CSCs are recruited by neoplastic cells mimicking the wound healing response [215]. These CAFs are located proximally to the tumor cells and display heightened activation markers like actin smooth muscle (aSMA), fibroblast activation protein (FAP), and fibroblast specific protein 1 (FSP1) [216,217]. The secretome of the CAF supports the stemness properties of cancers via growth factors like EGFR, IGFR, and PDGFR and help with recruitment of CSCs to the neoplastic site [218,219]. These specialized niches provide CSCs with the necessary factors to thrive in distant organs, allowing them to evade treatment and contribute to tumor progression. Understanding how CSCs interact with these pre-metastatic microenvironments is crucial for developing therapies aimed at blocking metastatic spread and improving patient outcomes.

In addition to EMT, CSCs exhibit remarkable plasticity that enables them to contribute to vasculogenic mimicry (VM), wherein they form vessel-like structures independent of endothelial cells [220]. CSCs can also integrate with endothelial cells to form mosaic vessels, facilitating tumor perfusion and dissemination [221]. These alternative vascularization mechanisms are associated with increased metastatic potential and therapy resistance, highlighting their fundamental role in supporting CSC survival and metastatic seeding in distant organs [222].

#### 2.6.2. Circulating Tumor Cells (CTCs)

CTCs are tumor-derived cells that detach from the primary tumor and enter the bloodstream or lymphatic system, playing a crucial role in metastasis [200,223,224,225]. Among them, a subset exhibits CSC-like properties, characterized by enhanced survival, therapy resistance, and metastatic potential. These CSC-like CTCs are highly adaptable, expressing markers such as CD44, ALDH1, and CD133, which contribute to their ability to resist apoptosis and persist in non-adherent conditions. Through EMT, these cells acquire invasive traits, allowing them to disseminate and colonize distant organs [210]. CTCs can be isolated from the blood of cancer patients and used as a biomarker for metastasis and prognosis. The survival advantage of CSC-like CTCs is driven by multiple mechanisms. The high expression of anti-apoptotic proteins (Bcl-2 and Bcl-xL) protects CSC-CTCs from cell death. They downregulate MHC-I expression and secrete immunosuppressive factors such as TGF-β, IL-10, and PD-L1 to escape immune surveillance, while interactions with platelets and neutrophils form a protective barrier against immune attack. Additionally, they upregulate drug efflux transporters (e.g., ABC transporters and MDR1) and enhance DNA repair pathways, making them highly resistant to chemotherapy and radiation. By expressing chemokine receptors such as CXCR4, CCR7, and CXCR1/2, CSC-like CTCs are guided to specific pre-metastatic niches where they can undergo the mesenchymal-to-epithelial transition (MET), reactivating the proliferative capacity and initiating tumor growth at secondary tumor sites. CSC-like CTCs preferentially colonize organ-specific microenvironment that provide survival and proliferation cues [223,224,225]. In the bone marrow, they interact with osteoblasts and stromal cells, often contributing to breast and prostate cancer metastases. In the lungs, integrins and the CXCL12/CXCR4 axis facilitate adhesion and invasion, while hepatic fibroblasts and stromal cells support CSC-like CTC survival in the liver. Upon reaching these niches, CSC-like CTCs can undergo MET, restoring their proliferative capacity and initiating tumor growth. Targeting the mechanisms that sustain CSC-like CTCs is essential for disrupting metastatic progression and improving therapeutic outcomes.

### 2.7. Therapeutic Strategies Targeting CSCs

Given the critical role of CSCs in tumorigenesis, metastasis, and drug resistance, targeting CSCs has emerged as a promising strategy for cancer therapy. Several therapeutic approaches have been developed to target CSCs, as is discussed below (Table 1).

#### 2.7.1. Targeting CSC-Specific Surface Markers

Targeting CSC-specific surface markers offers a promising strategy for eradicating CSCs and improving cancer treatment outcomes [128]. Several CSC-specific surface markers have been explored as therapeutic targets. CD44, highly expressed in solid tumors like breast, colon, and glioblastoma, regulates CSC self-renewal, migration, and the tumor microenvironment. Antibodies or small molecules targeting CD44 can block CSC self-renewal, inhibit tumor growth, and reduce the metastatic potential [226,227]. CD133 (Prominin-1), a membrane glycoprotein used as a key marker in brain and hematologic cancers, supports CSC maintenance [111]. Anti-CD133 monoclonal antibodies have shown promise in preclinical models by selectively targeting and eliminating CSCs without affecting normal tissue. High ALDH (Aldehyde Dehydrogenase) activity sustains stemness and therapy resistance in breast, colon, and liver cancers [115,226]. Inhibiting ALDH activity can reduce CSC self-renewal, promote differentiation, and increase sensitivity to chemotherapy. EpCAM (Epithelial Cell Adhesion Molecule) is a glycoprotein overexpressed in epithelial cancers, including breast, prostate, and colorectal cancers. It is implicated in cell adhesion and signaling and has been shown to be involved in the maintenance of CSC properties [117]. EpCAM-targeted therapies, including monoclonal antibodies and chimeric antigen receptor (CAR) T cells, have been explored to selectively target CSCs and eliminate tumor-initiating populations [228,229]. CD24 is a small glycosylphosphatidylinositol (GPI)-anchored membrane protein involved in cell signaling, migration, and adhesion. It is frequently expressed on CSCs in cancers such as breast, ovarian, and pancreatic cancers [226,230,231]. Targeting CD24 can lead to reduced CSC populations and improved responses to conventional therapies [232]. The Notch signaling pathway, crucial for CSC self-renewal, can be inhibited using γ-secretase inhibitors (e.g., DAPT), which has shown promise in preclinical studies by depleting CSCs and reducing tumor growth [233,234]. Integrins like α6β1 and αvβ3 are highly expressed on CSCs and play a role in stem cell migration, survival, and niche interactions [186]. Targeting integrin α6 or αvβ3 with monoclonal antibodies or small molecules can inhibit CSC migration and self-renewal, leading to reduced tumor progression and metastasis [235]. Lgr5 (Leucine-rich Repeat-containing G-protein Coupled Receptor 5), a stemness marker highly expressed in multiple tissues and CSCs, is being explored for CAR-T therapy [236]. CXCR4 is a chemokine receptor involved in cell migration and homing, especially in the context of CSC migration to distant sites. It is expressed on CSCs in various cancers, including breast and glioblastoma. Targeting CXCR4 with inhibitors can disrupt CSC migration and metastasis. CXCR4 inhibitors are being evaluated in clinical trials as adjuncts to chemotherapy or radiotherapy [237,238]. Collectively, these targeted therapies have the potential to reduce CSC populations, limit metastasis, and enhance the effectiveness of conventional treatments.

#### 2.7.2. Inhibiting CSC Signaling Pathways

Another approach to targeting CSCs is to inhibit the signaling pathways that regulate CSC self-renewal and survival, as discussed above. Several small molecule inhibitors and monoclonal antibodies have been developed to target these pathways, and some have shown promise in preclinical and clinical studies.

(1) Targeting the Wnt/β-Catenin Signaling Pathways

The Wnt/β-catenin signaling pathway is crucial for the self-renewal, proliferation, and survival of CSCs. Targeting Wnt/β-catenin signaling presents a promising strategy for eradicating CSCs and improving cancer treatment outcomes. Various small molecule inhibitors have been developed to target this pathway [138,239,240]. For example, Porcupine inhibitors (e.g., LGK974 and ETC-159) block Wnt secretion or its interaction with receptors, preventing Wnt signaling activation in CSCs [241,242,243]. LGK974 is currently being tested in clinical trials for multiple cancers. Tankyrase inhibitors (e.g., XAV939 and IWR-1) stabilize AXIN, leading to β-catenin degradation and Wnt pathway inhibition [244]. However, this may be less effective in tumors with mutations downstream of AXIN, such as CTNNB1 mutations. β-Catenin itself forms a complex with TCF/LEF proteins to activate CSC-related genes, and disrupting this complex can inhibit CSC function [245]. Inhibitors like ICG-001 and PRI-724 target β-catenin-mediated transcription, reducing CSC self-renewal [245,246,247]. PRI-724 is being tested for pancreatic cancer, leukemia, and solid tumors, and its combination with chemotherapy or immunotherapy shows enhanced treatment efficacy.

Additional strategies to target Wnt/β-catenin signaling include blocking Frizzled (FZD) receptors with monoclonal antibodies such as OMP-18R5 (vantictumab) and OMP-54F28, which prevent Wnt ligands from activating the pathway [138,248,249]. Vantictumab is undergoing clinical trials for solid tumors. Other approaches, such as OTSA101 and BC2059, disrupt Wnt-related CSC markers and β-catenin’s interaction with TBL1, leading to its degradation [138]. β-Catenin PROTACs (proteolysis-targeting chimeras) are also being developed for targeted protein degradation [250]. CSC plasticity often involves epigenetic modifications that sustain Wnt activity, and targeting epigenetic regulators can suppress CSC survival [203]. Histone deacetylase inhibitors (e.g., Vorinostat and Panobinostat) can reduce β-catenin stability and Wnt target gene expression, while DNA methyltransferase inhibitors (e.g., Decitabine and 5-Azacytidine) modulate Wnt pathway genes to suppress CSC properties [138,251]. Immunotherapy can enhance the anti-CSC effects of Wnt inhibitors, such as combining LGK974 with anti-PD-1 therapy. Additionally, Wnt-specific cancer vaccines and CAR-T cells engineered to target Wnt-dependent CSCs are under development as potential therapeutic options.

Some examples of clinical trials highlight both the promise and challenges of targeting the Wnt pathway in patients. WNT974, a Porcupine inhibitor, was evaluated in a phase I trial involving 94 patients with advanced solid tumors. The recommended dose was 10 mg daily, with dysgeusia as the most common adverse event. Although no objective tumor responses were observed per RECIST criteria, 16% of the patients achieved stable disease, and biomarker analyses confirmed effective Wnt pathway inhibition along with potential modulation of immune cell recruitment [235]. Another study assessed vantictumab in a phase I trial in combination with paclitaxel, a chemotherapy agent that stabilizes microtubules, for HER2-negative metastatic breast cancer. The combination yielded a 31.3% overall response rate and 68.8% clinical benefit rate, with a median progression-free survival of 3.8 months and overall survival of 12.7 months. While the regimen was generally well tolerated, fragility fractures occurred in several patients despite bone-protective measures, leading to the early termination of the study and limiting further development of vantictumab [232].

(2) Targeting the Hedgehog Signaling Pathways

The Hedgehog (Hh) signaling pathway is a key regulator of CSCs, promoting self-renewal, proliferation, survival, and therapy resistance [139]. The aberrant activation of this pathway is implicated in various cancers, including glioblastoma, pancreatic cancer, breast cancer, and leukemia. Targeting Hh signaling is a promising strategy to eliminate CSCs, overcome drug resistance, and prevent tumor relapse. Various approaches have been developed to target the Hh pathway. Smoothened (SMO) is a key activator of Hh signaling, and targeting SMO can prevent the downstream activation of GLI transcription factors. FDA-approved SMO inhibitors, such as Vismodegib (GDC-0449) and Sonidegib (LDE225), are approved for basal cell carcinoma, while Glasdegib (PF-04449913) is approved for acute myeloid leukemia [252,253,254,255]. However, mutations in SMO (e.g., D473H) and GLI activation independent of SMO through pathways like PI3K/Akt or TGF-β limit the efficacy of SMO inhibitors in targeting CSCs.

Direct GLI inhibition has emerged as a promising approach to overcome resistance to SMO inhibitors and suppress non-canonical Hedgehog activation. Inhibitors like GANT61, which block GLI1/GLI2 DNA binding, and Arsenic Trioxide (ATO), which targets GLI2 degradation, reduce CSC survival [256,257]. Itraconazole, an antifungal drug, has also shown potential in suppressing GLI signaling in CSCs [258]. Another strategy involves blocking Hedgehog ligand secretion to prevent both autocrine and paracrine signaling in CSCs. Hh ligand inhibitors such as Robotnikinin (which blocks SHH ligand binding to PTCH1), 5E1 (an Hh-neutralizing antibody), and CUR61414 (a small molecule antagonist) reduce CSC signaling in the tumor microenvironment and prevent paracrine activation from stromal cells [252]. Epigenetic modifications play a role in sustaining Hedgehog signaling in CSCs, and targeting these changes with histone deacetylase (HDAC) inhibitors like Vorinostat and Panobinostat, or DNA methyltransferase (DNMT) inhibitors such as 5-Azacytidine, can suppress CSC self-renewal [203,252,259]. Combining Hedgehog inhibitors with immunotherapy, including checkpoint inhibitors like anti-PD-1/PD-L1, enhances the anti-CSC effects by overcoming immune evasion. Additionally, Hedgehog-targeted cancer vaccines and CAR-T cells engineered to recognize Hedgehog-dependent CSC markers such as CD44 or LGR5 hold promise for further therapeutic applications.

Clinical evidence further illustrates both the potential and challenges of targeting this pathway. In a study of Sonidegib in patients with advanced basal cell carcinoma resistant to Vismodegib, most experience progressive disease or only brief stable disease, with no tumor shrinkage observed, suggesting that SMO mutations likely contributes to treatment failure and underscoring cross-resistance between SMO inhibitors and the need for alternative strategies [237]. In contrast, glasdegib is well tolerated in Japanese patients with advanced hematologic malignancies, showing manageable side effects and no dose-limiting toxicities at doses up to 100 mg daily. Encouragingly, one patient achieves complete remission in acute myeloid leukemia, accompanied by the suppression of GLI1 expression, supporting effective Hedgehog pathway inhibition at the downstream level [243].

(3) Targeting the Notch Signaling Pathways

The Notch signaling pathway plays a crucial role in CSC self-renewal, survival, differentiation, and resistance to therapy. Aberrant Notch activation drives tumorigenesis and CSC maintenance in multiple cancers, making it a promising therapeutic target [260,261]. Several strategies have been developed to target Notch signaling in CSCs [56,262]. γ-secretase inhibitors (GSIs) are one of the primary approaches to target the Notch pathway. GSIs block Notch cleavage, preventing the release of the Notch intracellular domain (NICD) and subsequent transcriptional activation. Drugs such as DAPT, MK-0752, RO4929097, and BMS-906024 are currently in clinical trials for solid tumors and leukemia. LY3039478 (CB-103) is a next-generation GSI with improved specificity [263]. The main advantage of GSIs is their broad inhibition of all Notch receptors, which can synergize with chemotherapy to overcome CSC resistance. However, a significant challenge is the gastrointestinal toxicity caused by inhibiting normal Notch function in intestinal stem cells.

Notch Ligand/Receptor Inhibitors work by blocking ligand–receptor interactions, preventing Notch activation in CSCs [264,265]. For instance, DLL4-targeting agents such as Demcizumab (OMP-21M18), a DLL4 antibody tested in pancreatic and lung cancers, and OMP-305B83, a bispecific antibody targeting DLL4 and VEGF, inhibit Notch1/Notch4 activation in CSCs. Jagged1/2-targeting agents, like OMP-59R5 (Tarextumab), block the Jagged-Notch interaction in CSCs [266]. These agents offer more specific inhibition of CSC-related Notch pathways and have reduced gastrointestinal toxicity compared to GSIs. However, resistance can develop through alternative Notch activation, and combining these agents with other therapies may be necessary to enhance their efficacy. Notch Transcription Inhibitors directly block NICD-dependent transcription, preventing the expression of Notch target genes [267,268]. Small molecule inhibitors like CB-103 and peptide inhibitors such as SAHM1, which prevent NICD from binding to DNA, are examples of this approach. These inhibitors selectively target Notch-driven gene expression in CSCs and overcome resistance caused by non-canonical Notch activation. However, challenges remain in drug delivery, the stability of peptide inhibitors, and the possibility of compensatory survival pathways in CSCs.

The epigenetic modulation of Notch signaling is another avenue for targeting CSCs [203]. Targeting CSC-specific epigenetic regulators can suppress Notch-driven transcription. Histone deacetylase (HDAC) inhibitors such as Vorinostat and Panobinostat reduce Notch target gene expression. DNA methyltransferase (DNMT) inhibitors like 5-Azacytidine (Decitabine) modify Notch-associated methylation, reactivating tumor suppressors. These epigenetic approaches can reverse CSC plasticity and prevent therapy resistance. However, they may cause off-target toxicity due to systemic epigenetic effects. Finally, combination therapy approaches have shown promise in improving the effectiveness of Notch inhibitors. GSIs combined with chemotherapy can overcome CSC-mediated chemoresistance, as seen in the combination of RO4929097 and Gemcitabine tested in pancreatic CSCs [56]. Notch inhibitors, when combined with PI3K/Akt inhibitors, target non-canonical Notch signaling, exemplified by Tarextumab and Everolimus in glioblastoma CSCs. Moreover, combining Notch inhibitors with immune checkpoint blockade can enhance immune targeting of CSCs, as demonstrated by DLL4 blockade and anti-PD-1 therapy, which improves T cell infiltration into CSC niches.

Several Notch-targeted therapies have progressed into clinical evaluation, offering insights into their therapeutic potential in CSC-associated cancers. In a phase I trial, MK-0752 is administered to pediatric patients with recurrent central nervous system tumors and is well tolerated at the recommended dose; however, liver enzyme elevations are the primary dose-limiting toxicity. While no tumor shrinkage is observed, the study confirms Notch pathway engagement and provides key safety data [252]. Similarly, Tarextumab is well tolerated in adults with advanced solid tumors, with some achieving stable disease alongside evidence of pathway inhibition [249]. In patients with adenoid cystic carcinoma (ACC) and other malignancies, CB-103 demonstrates manageable toxicity and biological activity, with nearly half of the patients achieving stable disease—including 58% of those with ACC—supporting further investigation in Notch-mutant tumors and combination therapy settings [251].

(4) Targeting the PI3K Signaling Pathways

The PI3K/Akt/mTOR pathway is a key regulator of CSC survival, self-renewal, metabolism, drug resistance, and metastasis [153,155,156]. The aberrant activation of this pathway sustains CSC maintenance, making it an attractive therapeutic target. Several inhibitors have been developed to target various components of this pathway. PI3K inhibitors, including Alpelisib (BYL719; FDA-approved for PIK3CA-mutant breast cancer), Buparlisib (BKM120), and PX-866, block PI3K activation and reduce CSC survival [269,270,271]. Additionally, PI3Kδ inhibitors such as Idelalisib target leukemia stem cells, while PI3Kβ inhibitors such as AZD8186 are effective in PTEN-deficient CSCs. These inhibitors have also shown promise in treating breast, colorectal, and brain tumors [151,153]. Akt inhibitors, such as Capivasertib (AZD5363) and Ipatasertib (GDC-0068) (used in PTEN-deficient CSCs), selectively block AKT and impair CSC self-renewal. AKT inhibition sensitizes CSCs to chemotherapy in glioblastoma and breast cancer. mTOR inhibitors targeting mTORC1/mTORC2 disrupt CSC metabolism and tumor growth [154]. Rapamycin, Everolimus (RAD001), and Temsirolimus (FDA-approved for renal and breast CSCs) target mTORC1, reducing CSC proliferation but failing to inhibit mTORC2, which can sustain CSCs. Dual mTORC1/2 inhibitors (INK128 and AZD8055) overcome [138,245] mTORC1-driven resistance. Simultaneous PI3K and mTOR inhibition (BEZ235, GDC-0980, and SF1126) prevents Akt feedback activation. Combination strategies, such as PI3K inhibitors (e.g., MK-2206) in combination with Wnt/Hedgehog/Notch inhibitors (e.g., Vismodegib), can enhance efficacy.

Clinical trials have underscored the therapeutic potential of PI3K inhibitors. In a phase I trial, Alpelisib combined with letrozole in ER+/HER2− metastatic breast cancer is well tolerated at 300 mg/day, with a 35% clinical benefit rate and greater response in PIK3CA-mutant patients; resistance is linked to FGFR1/2 amplifications and KRAS or TP53 mutations [255]. In another phase I trial, copanlisib shows good tolerability and activity in non-Hodgkin’s lymphoma, with all six follicular lymphoma patients responding, including one complete response [256]. The phase II DYNAMO study evaluates duvelisib in refractory indolent lymphoma, achieving a 47% response rate with a 10-month response duration and manageable toxicity, supporting its use in heavily pretreated patients [257].

#### 2.7.3. Inducing CSC Differentiation

Inducing differentiation in CSCs is a promising strategy to reduce their tumorigenic potential and enhance their susceptibility to conventional therapies. CSC maintenance is driven by embryonic signaling pathways, which can be manipulated to induce differentiation. Wnt/β-catenin inhibition disrupts CSC self-renewal by targeting β-catenin, with differentiation inducers like XAV939 (Tankyrase inhibitor) promoting differentiation in colon and glioblastoma CSCs, and PRI-724 (CBP/β-catenin inhibitor) inducing differentiation in leukemia and breast CSCs, as well as PI3K inhibitors (e.g., MK-2206) in combination with Wnt/Hedgehog/Notch inhibitors (e.g., Vismodegib) [138,245]. Similarly, Notch pathway modulation is critical, as high Notch signaling sustains CSC populations in glioblastoma, breast, and colon cancers. Notch inhibitors (γ-secretase inhibitors (GSIs)) such as RO4929097 and DAPT drive differentiation in glioblastoma, pancreatic, and breast CSCs [55,56]. Additionally, Hedgehog pathway inhibition prevents CSC quiescence and self-renewal, with Hedgehog inhibitors (Vismodegib and Sonidegib) promoting differentiation in glioblastoma, pancreatic, and medulloblastoma CSCs [139,252].

CSC fate is epigenetically regulated, making epigenetic modulators a potential strategy for differentiation therapy [203]. One common mechanism of CSC differentiation blockade is promoter methylation of differentiation genes, which can be reversed using DNA methyltransferase (DNMT) inhibitors like 5-Azacytidine and Decitabine, both of which induce differentiation in leukemia and glioblastoma CSCs [272,273]. Zebularine also reactivates differentiation genes in breast CSCs [274]. Since CSC differentiation requires an open chromatin state, histone deacetylase (HDAC) inhibitors such as Vorinostat, Panobinostat, and Trichostatin A promote differentiation in glioblastoma, neuroblastoma, and leukemia CSCs [275,276]. BET bromodomain inhibitors, which target MYC-driven CSC maintenance by disrupting chromatin remodeling, such as JQ1 and I-BET762, have also been shown to promote differentiation in leukemia, prostate, and glioblastoma CSCs [277,278].

Hormonal and metabolic reprogramming further offers opportunities for CSC differentiation therapy. Retinoic acid (ATRA and isotretinoin) promotes CSC differentiation via RAR/RXR signaling, with established success in acute promyelocytic leukemia (APL) and ongoing trials in glioblastoma, neuroblastoma, and breast CSCs [279,280]. Vitamin D analogues like 1,25-dihydroxyvitamin D3, Calcitriol, and Calcipotriol enhance differentiation in colon, breast, and leukemia CSCs [281,282], while thyroid hormones (T3/T4) have been shown to promote glioblastoma CSC differentiation [283]. Metabolic reprogramming also plays a role, as inhibiting fatty acid oxidation (FAO) with Etomoxir or Perhexiline forces CSC differentiation in glioblastoma and leukemia [284]. Glutaminase inhibitors (CB-839) promote CSC differentiation in glioblastoma and pancreatic cancer, while inducing oxidative metabolism using Metformin or mitochondrial uncouplers shifts CSCs away from glycolysis, promoting differentiation in breast and colon CSCs [285,286,287]. Finally, niche factors, such as hypoxia, cytokines, and stromal cells, regulate CSC fate and maintain CSCs in an undifferentiated state. Thus, HIF-1α inhibitors (Acriflavine, Digoxin, and PX-478) counteract hypoxia-driven CSC maintenance, while cytokines (IL-6, IFN-γ, and TNF-α) and Focal Adhesion Kinase (FAK) inhibitors (Defactinib) promote differentiation by altering the tumor microenvironment [102,157,159,176].

#### 2.7.4. Strategies to Overcome CSC Immune Evasion

CSCs possess multiple mechanisms to evade immune surveillance, enabling tumor persistence, metastasis, and therapy resistance. Overcoming immune evasion in CSCs is crucial for improving immunotherapy efficacy. Enhancing CSC immunogenicity and immune recognition can increase their susceptibility to immune attack. Strategies include restoring antigen presentation through epigenetic drugs like DNMT and HDAC inhibitors, which upregulate MHC-I expression and improve CSC recognition by T cells [288]. IFN-γ treatment increases antigen presentation in CSCs [289,290]. Cancer vaccine approaches, such as dendritic cell (DC) vaccines and mRNA vaccines encoding CSC-specific antigens (e.g., CD44 and ALDH1), are being explored to prime immune responses against CSCs [291,292].

The direct activation of immune cells against CSCs is another promising strategy. CSC-mediated immune suppression can be reversed by immune checkpoint inhibitors (ICIs). Anti-PD-1/PD-L1 therapies (e.g., pembrolizumab and nivolumab) reactivate T cells, counteracting CSC-induced immune evasion [293,294]. Additionally, CSCs evade macrophage-mediated phagocytosis by expressing CD47, a “don’t eat me” signal. CD47 blockade (e.g., Magrolimab and TTI-621) disrupts this mechanism, enhancing macrophage-driven clearance of CSCs and promoting their phagocytosis [295]. Additionally, chimeric antigen receptor (CAR) T cell therapy engineered to target CSC markers (CD133, EpCAM, and CD44) has shown potential, with dual-targeting CAR-T approaches (e.g., CD133 + PD-L1) designed to overcome immune evasion [296,297]. NK cell-based therapies, including adoptive NK cell transfer and NKG2D agonists, enhance CSC cytotoxicity, while IL-15 and IL-21 treatments further boost NK cell function [298,299]. Moreover, macrophage polarization to an M1 phenotype using CSF-1R inhibitors (Pexidartinib) and TLR agonists can shift tumor-associated macrophages from a pro-tumorigenic to an anti-tumorigenic state, thereby increasing immune-mediated CSC clearance [300,301,302].

Targeting the CSC-supportive microenvironment weakens immune evasion and enhances CSC elimination. The immunosuppressive niche is maintained by factors such as TGF-β signaling, hypoxia, and CXCR4/CXCL12-mediated immune cell recruitment [181]. TGF-β inhibitors (Galunisertib and Fresolimumab) reduce regulatory T cell (Treg) infiltration and enhance cytotoxic T lymphocyte (CTL) responses, while HIF-1α inhibitors (Acriflavine and PX-478) counteract hypoxia-induced immune resistance [303,304]. Disrupting the CXCR4/CXCL12 axis with inhibitors like Plerixafor improves T cell infiltration into CSC niches [128]. Combination strategies, such as anti-PD-1 therapy with CSC differentiation inducers (e.g., ATRA), CAR-T cells with checkpoint blockade, and NK cell therapy with metabolic reprogramming (e.g., Metformin), offer synergistic approaches to eliminating CSCs and preventing immune escape.

#### 2.7.5. Strategies to Overcome ABC Transporter-Mediated Resistance in CSCs

ABC transporters, such as P-glycoprotein (P-gp), breast cancer resistance protein (BCRP), and multidrug resistance protein 1 (MRP1), play a significant role in conferring chemotherapy resistance in CSCs [95]. These transporters actively efflux chemotherapeutic agents, reducing drug efficacy and contributing to tumor relapse. Developing strategies to circumvent this resistance could significantly enhance the effectiveness of treatments for CSC-targeted therapies. One approach to counteract this resistance is the use of ABC transporter inhibitors, such as Tariquidar (P-gp inhibitor), Ko143 (BCRP inhibitor), and MK-571 (MRP1 inhibitor) [305,306]. However, many ABC transporter inhibitors have failed in clinical trials due to toxicity and off-target effects, limiting their widespread application. An alternative strategy is combination therapy, which involves using ABC transporter inhibitors alongside chemotherapy to enhance drug retention in CSCs. For example, Tariquidar has been combined with Doxorubicin to overcome MDR1-mediated resistance, improving treatment efficacy [307]. This approach seeks to maximize the impact of chemotherapy while minimizing systemic toxicity. Additionally, targeting the expression of ABC transporters through RNA interference (RNAi), CRISPR-based gene editing, or epigenetic modulation (e.g., DNA methylation inhibitors or histone deacetylase inhibitors) offers potential ways to reduce transporter activity and enhance drug accumulation in CSCs.

Another promising avenue is the use of nanoparticle-based drug delivery systems, which can bypass ABC transporter activity by enhancing drug uptake in CSCs. Liposomes and polymeric micelles encapsulating chemotherapeutic agents can improve drug stability and targeted delivery, minimizing efflux by ABC transporters [308,309]. Furthermore, strategies targeting the tumor microenvironment, such as inhibiting hypoxia-induced ABC transporter expression or reprogramming CSC-supportive signaling pathways (e.g., Wnt, Notch, and Hedgehog), may further sensitize CSCs to chemotherapy. Immunotherapy approaches, including CAR-T cell therapy and immune checkpoint inhibitors, are also being explored to eliminate CSCs directly, bypassing the need to counteract ABC transporter-mediated drug resistance.

#### 2.7.6. Targeting DNA Repair in CSCs for Therapy

Targeting DNA repair mechanisms in CSCs represents a promising therapeutic strategy, as CSCs often exhibit resistance to conventional therapies like chemotherapy and radiation due to their enhanced DNA repair capacity [310,311]. These cells rely on multiple DNA repair pathways, including homologous recombination (HR), non-homologous end joining (NHEJ), base excision repair (BER), and mismatch repair (MMR), to maintain genomic stability and prevent the accumulation of lethal mutations. Disrupting these pathways can sensitize CSCs to DNA-damaging therapies and reduce tumor recurrence. For example, HR inhibitors targeting BRCA1/2 or RAD51 can impair CSCs’ ability to repair double-strand breaks (DSBs), making them more vulnerable to DNA damage [312]. Similarly, PARP inhibitors can induce synthetic lethality in CSCs by preventing the repair of single-strand breaks, leading to persistent DNA damage and cell death [313].

Beyond direct DNA repair inhibition, targeting the DNA damage response (DDR) offers another effective strategy. CSCs often exhibit an enhanced DDR, relying on key regulators such as ATM, ATR, and CHK1 to counteract replication stress and oxidative damage [314,315]. Inhibiting DDR components like ATR (with VE-821) or CHK1 (with prexasertib) can prevent CSCs from efficiently responding to DNA damage, thereby increasing their susceptibility to genotoxic therapies. ATM inhibitors further compromise CSCs’ ability to repair DSBs, sensitizing them to radiation and chemotherapy. Additionally, targeting replication stress-related proteins such as Replication Protein A (RPA) and CDC25 can exacerbate genomic instability in CSCs, leading to selective cell death [316].

Combining DNA repair inhibitors with conventional DNA-damaging therapies enhances their therapeutic efficacy. Chemotherapy and radiotherapy, which induce DNA damage, can be potentiated by co-treatment with DNA repair inhibitors to prevent CSCs from effectively repairing treatment-induced lesions. For example, PARP inhibitors combined with cisplatin or temozolomide create a synergistic effect, leading to sustained DNA damage and increased tumor regression [317]. Additionally, integrating DNA repair inhibitors with targeted therapies that disrupt oncogenic signaling pathways, such as PI3K/Akt/mTOR inhibitors, can further weaken CSCs by simultaneously impairing their survival and DNA repair capacity [318]. These combination strategies hold great promise in overcoming CSC resistance and improving long-term treatment outcomes.

#### 2.7.7. Targeting Quiescent CSCs for Therapy

CSCs exist in both quiescent and proliferative states, with quiescent CSCs being particularly resistant to conventional therapies such as chemotherapy and radiation, which primarily target rapidly dividing cells. A major strategy to overcome this resistance involves inducing CSCs to exit dormancy, making them more susceptible to treatment. This “wake-up” strategy includes targeting dormancy regulators like p21 and p27, whose inhibition forces CSCs into the cell cycle [319]. Additionally, CDK4/6 inhibitors such as Palbociclib, when combined with chemotherapy, can push CSCs into proliferation [320]. Pathways like Notch and TGF-β, which reinforce dormancy, can also be disrupted using inhibitors like DAPT, RO4929097, and Galunisertib to promote CSC cycling [321,322]. Another approach is modulating Wnt/β-catenin signaling, where Wnt agonists like Wnt3A and R-spondins can stimulate CSC division and enhance their sensitivity to chemotherapy [138,245,247]. By driving CSCs into an active state, these strategies aim to reduce their ability to evade therapy and contribute to tumor relapse.

Alternatively, directly targeting quiescent CSCs through their unique survival mechanisms presents another therapeutic avenue. Since quiescent CSCs rely on oxidative phosphorylation (OXPHOS) rather than glycolysis, metabolic inhibitors like Metformin and IACS-10759 can selectively kill these cells [323,324]. Additionally, quiescent CSCs depend on autophagy for survival, making autophagy inhibitors such as Chloroquine and Spautin-1 effective in disrupting their maintenance [325,326]. The CSC microenvironment also plays a crucial role in their dormancy and survival, with hypoxia-targeting therapies like PX-478 and CXCR4 inhibitors like Plerixafor mobilizing CSCs out of protective niches [159,176]. Furthermore, immunotherapy approaches such as CAR-T and NK cell therapy can target CSC-specific antigens, while immune checkpoint inhibitors like Pembrolizumab and CD47 inhibitors enhance immune clearance [174,291,296]. Combining these strategies—such as pairing CDK4/6 inhibitors with chemotherapy, OXPHOS inhibitors with immunotherapy, or hypoxia inhibitors with radiation—offers a multifaceted approach to eliminating CSCs and reducing tumor recurrence.

## 3. Normal Stem Cells in Cancer Therapy

**Figure 4:**
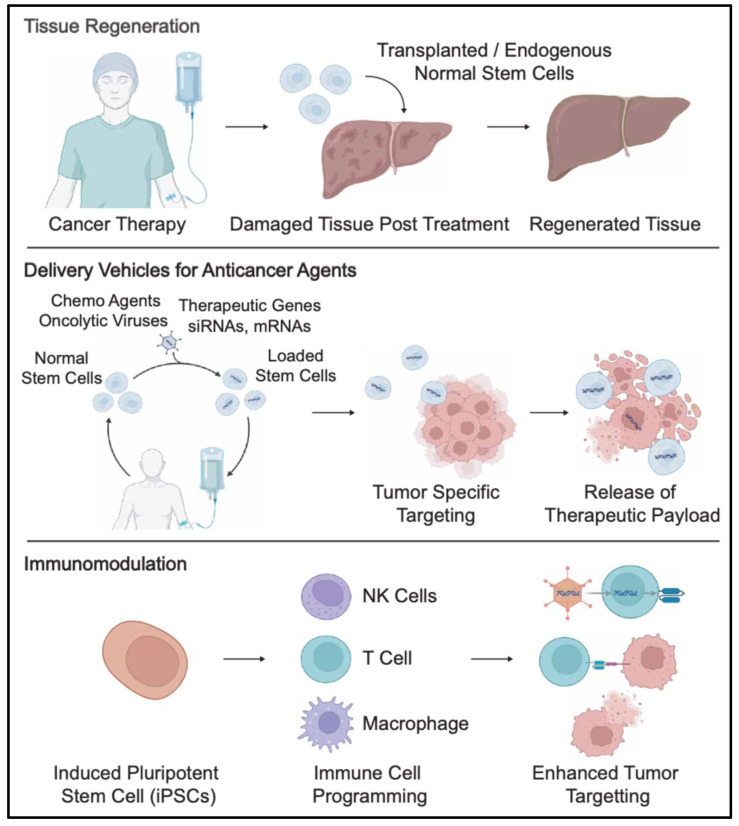
Normal stem cells in cancer therapy. A schematic depicting the major opportunities for cancer therapies using stem cells. First, tissue regeneration may be stimulated by stem cell populations. Second, stem cells can serve as targeted drug delivery vehicles directed toward tumor cells. Lastly, immune programming of stem cells offers a potential strategy for selectively targeting and eliminating tumor cells.

Normal stem cells play a critical role in cancer therapy due to their regenerative potential, their ability to differentiate into specialized cell types, and their capacity to modulate the tumor microenvironment [327,328]. These properties allow them to contribute to tissue repair, enhance immune responses, and serve as delivery vehicles for anticancer agents, making them promising candidates for innovative cancer treatments (Figure 4).

### 3.1. Tissue Regeneration

Normal stem cells are essential in tissue regeneration following cancer treatments like chemotherapy, radiation, or surgery. These therapies often result in significant tissue damage, but stem cells can aid in healing and restoring function. HSPCs are used in bone marrow transplants to regenerate blood cells and restore the immune system, red blood cells, and platelets after chemotherapy or radiation [329]. Epidermal stem cells help regenerate skin after radiation, accelerating wound healing and restoring barrier function, while oral mucosal stem cells can treat mucositis caused by head and neck cancer treatments [330,331]. Neural stem cells (NSCs) are being explored for their potential to regenerate brain tissue and repair neuronal damage after cancer treatment-related neurotoxicity, and mesenchymal stem cells (MSCs) can repair bone and muscle damage caused by surgery or radiation [332,333]. Stem cells also play a key role in regenerating vital organs and enhancing wound healing [331]. In cases of liver or kidney damage from cancer therapy, stem cells can differentiate into hepatocytes or renal cells to help restore function [334]. Additionally, stem cells promote wound healing after tumor resection by stimulating cell proliferation, collagen formation, and angiogenesis [335]. They can also help prevent scarring and fibrosis, common side effects of radiation therapy, by modulating the wound healing process. This regenerative capacity is crucial for improving recovery and quality of life in cancer patients.

### 3.2. Delivery Vehicles for Anticancer Agents

Normal stem cells are being increasingly investigated as delivery vehicles for anticancer agents due to their natural ability to home to tumor sites, a phenomenon known as “tumor tropism” [336,337]. This unique characteristic allows stem cells, such as MSCs and NSCs, to migrate toward and specifically target cancerous tissues, making them ideal candidates for enhancing drug delivery. These stem cells can be genetically engineered to express specific receptors, surface markers, or enzymes that further enhance their ability to precisely target tumor cells. By loading these cells with various therapeutic agents, including chemotherapy drugs, oncolytic viruses, therapeutic genes (e.g., cytokines and pro-apoptotic factors), and RNA molecules (e.g., siRNA and mRNA), stem cells can deliver treatments directly to the tumor while minimizing exposure to healthy tissues [338,339,340].

Once at the tumor site, stem cells can release their therapeutic payloads through passive diffusion, cellular secretion, or stimuli-responsive mechanisms (such as pH-sensitive coatings or enzyme-triggered release systems). The leaky blood vessels typically found in tumors further facilitate stem cell penetration, ensuring that the treatment is delivered directly to the cancerous area. MSCs are particularly studied for their ability to home to tumors in various cancers, including breast cancer, lung cancer, and glioblastoma. NSCs, with their natural affinity for central nervous system tissues, are primarily used for brain tumor therapies. Additionally, iPSCs hold promise for being differentiated into various cell types, offering a versatile option for targeted drug delivery. Preclinical research has shown the potential of MSCs in delivering oncolytic viruses or immune-modulating agents to breast cancer tumors, and they are being explored for use in liver cancer treatment as well. The ability of stem cells to migrate directly to the tumor site enhances the precision of anticancer therapies, improving treatment efficacy while reducing damage to healthy tissues. Furthermore, stem cells can survive in the body for extended periods, allowing for the continuous release of therapeutic agents, thereby prolonging therapeutic effects and minimizing side effects compared to traditional chemotherapy.

### 3.3. Stem Cells in Immunotherapy

Normal stem cells possess great potential in immunotherapy, leveraging their ability to modulate the immune system and strengthen the body’s natural defenses against cancer. iPSCs can be reprogrammed into immune cells like T cells, NK cells, and macrophages, which can be genetically engineered to target cancer cells more effectively [170,171,295,297]. These cells, including CAR-T cells, are enhanced to attack tumors with greater specificity. HSPCs play a critical role in immune system regeneration, particularly following chemotherapy or radiation, by restoring blood and immune cells, and can be combined with immunotherapy to better target residual cancer cells [329,336]. Additionally, MSCs are studied for their ability to modulate the TME, influencing immune cells and enhancing anti-tumor immunity by reducing immunosuppressive signals [333,341]. Engineered stem cells are also being used in targeted anti-tumor therapies by producing cytokines like IL-2 or IFN-α at tumor sites to boost the immune response while minimizing systemic side effects [289,292]. Stem cells’ regenerative properties are crucial in repairing tissue damage caused by aggressive immunotherapies, restoring normal tissue function and reducing inflammation. Moreover, combining stem cells with immune checkpoint inhibitors (e.g., PD-1 or CTLA-4 inhibitors) can enhance the immune system’s response against tumors, making them more effective. Stem cell-based therapies can also be integrated with traditional treatments such as chemotherapy or radiation to regenerate the immune system, improving the overall efficacy of cancer treatment. These strategies highlight the growing role of stem cells in advancing cancer immunotherapy, offering innovative solutions for more targeted and effective treatments.

## 4. Challenges and Future Directions

The transformation of normal stem cells into CICs is a multifaceted process driven by genetic mutations, epigenetic modifications, and changes in the surrounding microenvironment [203]. This transformation often marks the onset of cancer, and understanding these mechanisms lays the foundation for developing therapies that target the root causes of tumor initiation. Future research should aim to identify early biomarkers of stem cell transformation and design therapies capable of preventing malignant conversion or selectively eliminating CSCs, thus improving patient outcomes. An alternative pathway to tumorigenesis involves the dedifferentiation or reprogramming of differentiated cells into CICs, complementing the traditional stem cell-driven model. Though less explored, this process is equally important for understanding tumor initiation and evolution. Investigating the molecular drivers of dedifferentiation could lead to therapies that prevent or reverse this process, offering potential strategies for treating aggressive and therapy-resistant cancers. Another key aspect of cancer progression is EMT, a process that drives tumor invasion, metastasis, drug resistance, and stemness acquisition. The connection between EMT and CSCs is complex, and unraveling this relationship could lead to targeted therapies that prevent metastatic spread and enhance patient survival. Additionally, CSCs employ various immune evasion strategies, including antigen downregulation, the activation of immune checkpoints, and the recruitment of immunosuppressive cells. Understanding these mechanisms could open new opportunities for developing immunotherapies that specifically target CSCs, reducing relapse and improving long-term survival for cancer patients.

Despite advancements, challenges remain in CSC research. A major obstacle is the heterogeneity of CSCs, which complicates the identification of universal markers and the design of effective targeted therapies. Advances in single-cell sequencing could help decipher the heterogeneity of CSCs and identify subpopulations that might be resistant to therapy. The early detection of mutations in high-risk individuals, potentially through liquid biopsies and multi-omics approaches, could help identify stem cells at risk of transformation. CRISPR-based technologies offer the potential to correct oncogenic mutations in predisposed stem cell populations, preventing the initiation of cancer. Senescence-inducing therapies may also play a role in preventing the accumulation of pre-malignant stem cells. CSCs heavily rely on the tumor microenvironment for their maintenance and function. Therefore, exploring combination therapies that target both CSCs and the tumor microenvironment may be necessary to eradicate CSCs effectively.

Additionally, addressing safety concerns surrounding stem cell-based therapies is crucial. Potential risks include uncontrolled proliferation, differentiation into unintended cell types, immune rejection of allogeneic (donor-derived) stem cells, and tumor-promoting effects such as enhanced angiogenesis, immune suppression, and metastasis. Optimizing stem cell engineering—particularly strategies for the precise control of drug or gene release—will be essential for developing effective cancer treatments. Furthermore, the clinical use of normal stem cells raises significant ethical and safety concerns. For example, the generation of human embryonic stem cell (hESC) lines typically requires the destruction of human embryos, which has led to considerable ethical debate. To address this, researchers have developed methods to isolate cells from earlier embryonic stages using single blastomeres, like techniques employed in preimplantation genetic diagnosis [326]. The transplantation of hESCs has also been associated with teratoma formation in immunodeficient mice [313]. As a result, there is growing consensus to avoid the use of hESCs in favor of autologous stem cells or induced pluripotent stem cells (iPSCs), which circumvent ethical concerns and reduce the risk of immune incompatibility. Nevertheless, rigorous testing is necessary to maximize therapeutic benefits while minimizing potential risks for stem cell-based therapies.

Moreover, CSCs exhibit significant plasticity, which allows them to escape treatments and repopulate tumors. Therapy resistance mechanisms, such as quiescence and efflux pump activity, present additional challenges that require further investigation. Quiescent CSCs, which are dormant and resistant to therapy, pose a significant barrier in cancer treatment as well. Reactivating dormant CSCs or directly targeting their survival mechanisms may provide a strategy to overcome therapy resistance and improve long-term outcomes for patients.

## 5. Conclusions

Stem cells, particularly CSCs, are key drivers of cancer initiation, progression, and therapy resistance. Targeting CSCs through surface markers, signaling pathways, and differentiation has shown promise for cancer treatment. Moreover, normal stem cells are being explored for their potential in tissue regeneration and immunomodulation in cancer therapy. However, challenges like CSC heterogeneity, the tumor microenvironment, and risks associated with normal stem cells persist. Future research should deepen our understanding of CSC biology, improve CSC-targeting strategies, and optimize combination therapies to enhance cancer treatment outcomes and reduce disease burden.

## Figures and Tables

**Table 1:** Key pathways associated with CSCs and their therapeutic strategies.

Pathway	Key Components	Functions in CSCs	Therapeutic Strategies
**WNT/β-CATENIN**	Wnt ligands Frizzled (FZD) receptors LRP5/6 β-catenin TCF/LEF	Maintains CSC self-renewal Promotes therapy resistance Enhances EMT and metastasis	Porcupine inhibitors (LGK974, ETC-159) β-catenin inhibitors (PRI-724, ICG-001) Frizzled receptor blockers (OMP-18R5, vantictumab)
**HEDGEHOG (HH)**	SHH/IHH/DHH ligands Patched (PTCH) Smoothened (SMO) GLI1/2	Regulates CSC survival Enhances drug resistance and EMT Drives tumor progression	SMO inhibitors (Vismodegib, Sonidegib, Glasdegib) GLI inhibitors (GANT61, Arsenic Trioxide) Hh ligand inhibitors (5E1, Robotnikinin)
**NOTCH**	Notch1-4 receptors Jagged/Delta ligands NICD (Notch Intracellular Domain)	Maintains CSC populations Increases chemoresistance Promotes tumor angiogenesis and immune evasion	γ-secretase inhibitors (DAPT, MK-0752, RO4929097) Notch ligand inhibitors (Demcizumab, Tarextumab) Notch transcription inhibitors (CB-103, SAHM1)
**PI3K/AKT/MTOR**	PI3K AKT mTORC1/mTORC2 PTEN	Regulates CSC metabolism and survival Enhances drug resistance via ABC transporters Promotes tumor invasion and metastasis	PI3K inhibitors (Alpelisib, Buparlisib, PX-866) AKT inhibitors (Capivasertib, MK-2206, Ipatasertib) mTOR inhibitors (Rapamycin, Everolimus, AZD8055)
**TGF-β**	TGF-β ligands TGF-β receptors SMAD2/3/4	Drives EMT and metastasis Induces therapy resistance and immune suppression	TGF-β inhibitors (Galunisertib, Fresolimumab) SMAD inhibitors
**JAK/STAT**	IL-6 JAK1/2 STAT3/5	Promotes inflammation-induced CSC expansion Enhances immune evasion	JAK inhibitors (Ruxolitinib, Tofacitinib) STAT3 inhibitors (WP1066, Stattic)
**HIPPO/YAP**	MST1/2 LATS1/2 YAP/TAZ	Enhances CSC self-renewal Regulates stemness and drug resistance	YAP inhibitors (Verteporfin, CA3)
**NF-KB**	IKK IκB p65/RelA	Enhances CSC survival and immune evasion	IKK inhibitors (Bortezomib, Bay 11-7082)

## Abbreviations

The following abbreviations are used in this manuscript:

ACC	Adenoid cystic carcinoma
ALDH	Aldehyde dehydrogenase
AML	Acute myeloid leukemia
aSMA	actin smooth muscle
BER	Base excision repair
CAFs	Cancer-associated fibroblasts
CAR-T	Chimeric antigen receptor T cells
CICs	Cancer-initiating cells
CRCSCs	Colorectal cancer stem cells
CSCs	Cancer stem cells
CTCs	Circulating tumor cells
DSBs	Double-strand breaks
ECM	Extracellular matrix
EMT	Epithelial–mesenchymal transition
FAK	Focal adhesion kinase
FAP	Fibroblast activation protein
FSP1	Fibroblast specific protein 1
GSCs	Glioma stem cells
hESC	Human embryonic stem cell
HR	Homologous recombination
HSPCs	Hematopoietic stem and progenitor cells
iPSCs	Induced pluripotent stem cells
LSCs	Leukemia stem cells
MACS	Magnetic-activated cell sorting
MDSCs	Myeloid-derived suppressor cells
MMR	Mismatch repair
MSCs	Mesenchymal stem cells
NHEJ	Non-homologous end joining
NK	Natural Killer
NSCLC	Non-small cell lung cancer
NSCs	Neural stem cells
PDAC	Pancreatic ductal adenocarcinoma
RNAi	RNA interference
TAMs	Tumor-associated macrophages
TME	Tumor microenvironment
TNBC	Triple-negative breast cancer
VM	Vasculogenic mimicry

## References

[1] Quintana E., Shackleton M., Sabel M.S., Fullen D.R., Johnson T.M., Morrison S.J. (2008). Efficient tumour formation by single human melanoma cells. Nature.

[2] Kelly P.N., Dakic A., Adams J.M., Nutt S.L., Strasser A. (2007). Tumor growth need not be driven by rare cancer stem cells. Science.

[3] Clarke M.F., Dick J.E., Dirks P.B., Eaves C.J., Jamieson C.H., Jones D.L., Visvader J., Weissman I.L., Wahl G.M. (2006). Cancer stem cells--perspectives on current status and future directions: AACR Workshop on cancer stem cells. Cancer Res..

[4] Hope K.J., Jin L., Dick J.E. (2004). Acute myeloid leukemia originates from a hierarchy of leukemic stem cell classes that differ in self-renewal capacity. Nat. Immunol..

[5] Loh J.J., Ma S. (2024). Hallmarks of cancer stemness. Cell Stem Cell.

[6] Pardal R., Clarke M.F., Morrison S.J. (2003). Applying the principles of stem-cell biology to cancer. Nat. Rev. Cancer.

[7] Reya T., Morrison S.J., Clarke M.F., Weissman I.L. (2001). Stem cells, cancer, and cancer stem cells. Nature.

[8] Hancock J.F. (2003). Ras proteins: Different signals from different locations. Nat. Rev. Mol. Cell Biol..

[9] Voice J.K., Klemke R.L., Le A., Jackson J.H. (1999). Four human ras homologs differ in their abilities to activate Raf-1, induce transformation, and stimulate cell motility. J. Biol. Chem..

[10] Sinn E., Muller W., Pattengale P., Tepler I., Wallace R., Leder P. (1987). Coexpression of MMTV/v-Ha-ras and MMTV/c-myc genes in transgenic mice: Synergistic action of oncogenes in vivo. Cell.

[11] Li S., Balmain A., Counter C.M. (2018). A model for RAS mutation patterns in cancers: Finding the sweet spot. Nat. Rev. Cancer.

[12] Ligresti G., Militello L., Steelman L.S., Cavallaro A., Basile F., Nicoletti F., Stivala F., McCubrey J.A., Libra M. (2009). PIK3CA mutations in human solid tumors: Role in sensitivity to various therapeutic approaches. Cell Cycle.

[13] Vasan N., Razavi P., Johnson J.L., Shao H., Shah H., Antoine A., Ladewig E., Gorelick A., Lin T.Y., Toska E. (2019). Double PIK3CA mutations in cis increase oncogenicity and sensitivity to PI3Kα inhibitors. Science.

[14] Van Keymeulen A., Lee M.Y., Ousset M., Brohee S., Rorive S., Giraddi R.R., Wuidart A., Bouvencourt G., Dubois C., Salmon I. (2015). Reactivation of multipotency by oncogenic PIK3CA induces breast tumour heterogeneity. Nature.

[15] Madsen R.R., Knox R.G., Pearce W., Lopez S., Mahler-Araujo B., McGranahan N., Vanhaesebroeck B., Semple R.K. (2019). Oncogenic PIK3CA promotes cellular stemness in an allele dose-dependent manner. Proc. Natl. Acad. Sci. USA.

[16] Little C.D., Nau M.M., Carney D.N., Gazdar A.F., Minna J.D. (1983). Amplification and expression of the c-myc oncogene in human lung cancer cell lines. Nature.

[17] Lee K.M., Giltnane J.M., Balko J.M., Schwarz L.J., Guerrero-Zotano A.L., Hutchinson K.E., Nixon M.J., Estrada M.V., Sanchez V., Sanders M.E. (2017). MYC and MCL1 Cooperatively Promote Chemotherapy-Resistant Breast Cancer Stem Cells via Regulation of Mitochondrial Oxidative Phosphorylation. Cell Metab..

[18] Dang C.V. (2012). MYC on the path to cancer. Cell.

[19] Chen X., Zhang T., Su W., Dou Z., Zhao D., Jin X., Lei H., Wang J., Xie X., Cheng B. (2022). Mutant p53 in cancer: From molecular mechanism to therapeutic modulation. Cell Death Dis..

[20] Hollstein M., Sidransky D., Vogelstein B., Harris C.C. (1991). p53 mutations in human cancers. Science.

[21] Liu Y., Su Z., Tavana O., Gu W. (2024). Understanding the complexity of p53 in a new era of tumor suppression. Cancer Cell.

[22] Levine A.J., Oren M. (2009). The first 30 years of p53: Growing ever more complex. Nat. Rev. Cancer.

[23] Banin S., Moyal L., Shieh S., Taya Y., Anderson C.W., Chessa L., Smorodinsky N.I., Prives C., Reiss Y., Shiloh Y. (1998). Enhanced phosphorylation of p53 by ATM in response to DNA damage. Science.

[24] Li T., Kon N., Jiang L., Tan M., Ludwig T., Zhao Y., Baer R., Gu W. (2012). Tumor suppression in the absence of p53-mediated cell-cycle arrest, apoptosis, and senescence. Cell.

[25] Baslan T., Morris J.P.T., Zhao Z., Reyes J., Ho Y.J., Tsanov K.M., Bermeo J., Tian S., Zhang S., Askan G. (2022). Ordered and deterministic cancer genome evolution after p53 loss. Nature.

[26] Sun X., Klingbeil O., Lu B., Wu C., Ballon C., Ouyang M., Wu X.S., Jin Y., Hwangbo Y., Huang Y.H. (2023). BRD8 maintains glioblastoma by epigenetic reprogramming of the p53 network. Nature.

[27] Burkhart D.L., Sage J. (2008). Cellular mechanisms of tumour suppression by the retinoblastoma gene. Nat. Rev. Cancer.

[28] Huang H.J., Yee J.K., Shew J.Y., Chen P.L., Bookstein R., Friedmann T., Lee E.Y., Lee W.H. (1988). Suppression of the neoplastic phenotype by replacement of the RB gene in human cancer cells. Science.

[29] Li F., Kitajima S., Kohno S., Yoshida A., Tange S., Sasaki S., Okada N., Nishimoto Y., Muranaka H., Nagatani N. (2019). Retinoblastoma Inactivation Induces a Protumoral Microenvironment via Enhanced CCL2 Secretion. Cancer Res..

[30] Calo E., Quintero-Estades J.A., Danielian P.S., Nedelcu S., Berman S.D., Lees J.A. (2010). Rb regulates fate choice and lineage commitment in vivo. Nature.

[31] Vidotto T., Melo C.M., Lautert-Dutra W., Chaves L.P., Reis R.B., Squire J.A. (2023). Pan-cancer genomic analysis shows hemizygous PTEN loss tumors are associated with immune evasion and poor outcome. Sci. Rep..

[32] Lee Y.R., Chen M., Pandolfi P.P. (2018). The functions and regulation of the PTEN tumour suppressor: New modes and prospects. Nat. Rev. Mol. Cell Biol..

[33] Wang S., Gao J., Lei Q., Rozengurt N., Pritchard C., Jiao J., Thomas G.V., Li G., Roy-Burman P., Nelson P.S. (2003). Prostate-specific deletion of the murine Pten tumor suppressor gene leads to metastatic prostate cancer. Cancer Cell.

[34] Zhang Y., Kwok-Shing Ng P., Kucherlapati M., Chen F., Liu Y., Tsang Y.H., de Velasco G., Jeong K.J., Akbani R., Hadjipanayis A. (2017). A Pan-Cancer Proteogenomic Atlas of PI3K/AKT/mTOR Pathway Alterations. Cancer Cell.

[35] Nishiyama A., Nakanishi M. (2021). Navigating the DNA methylation landscape of cancer. Trends Genet..

[36] Herman J.G., Merlo A., Mao L., Lapidus R.G., Issa J.P., Davidson N.E., Sidransky D., Baylin S.B. (1995). Inactivation of the CDKN2/p16/MTS1 gene is frequently associated with aberrant DNA methylation in all common human cancers. Cancer Res..

[37] Hansen K.D., Timp W., Bravo H.C., Sabunciyan S., Langmead B., McDonald O.G., Wen B., Wu H., Liu Y., Diep D. (2011). Increased methylation variation in epigenetic domains across cancer types. Nat. Genet..

[38] Spencer D.H., Russler-Germain D.A., Ketkar S., Helton N.M., Lamprecht T.L., Fulton R.S., Fronick C.C., O’Laughlin M., Heath S.E., Shinawi M. (2017). CpG Island Hypermethylation Mediated by DNMT3A Is a Consequence of AML Progression. Cell.

[39] Marsolier J., Prompsy P., Durand A., Lyne A.M., Landragin C., Trouchet A., Bento S.T., Eisele A., Foulon S., Baudre L. (2022). H3K27me3 conditions chemotolerance in triple-negative breast cancer. Nat. Genet..

[40] Gollner S., Oellerich T., Agrawal-Singh S., Schenk T., Klein H.U., Rohde C., Pabst C., Sauer T., Lerdrup M., Tavor S. (2017). Loss of the histone methyltransferase EZH2 induces resistance to multiple drugs in acute myeloid leukemia. Nat. Med..

[41] Yamagishi M., Kuze Y., Kobayashi S., Nakashima M., Morishima S., Kawamata T., Makiyama J., Suzuki K., Seki M., Abe K. (2024). Mechanisms of action and resistance in histone methylation-targeted therapy. Nature.

[42] Bagchi A., Papazoglu C., Wu Y., Capurso D., Brodt M., Francis D., Bredel M., Vogel H., Mills A.A. (2007). CHD5 is a tumor suppressor at human 1p36. Cell.

[43] Mashtalir N., D’Avino A.R., Michel B.C., Luo J., Pan J., Otto J.E., Zullow H.J., McKenzie Z.M., Kubiak R.L., St Pierre R. (2018). Modular Organization and Assembly of SWI/SNF Family Chromatin Remodeling Complexes. Cell.

[44] Wong A.K., Shanahan F., Chen Y., Lian L., Ha P., Hendricks K., Ghaffari S., Iliev D., Penn B., Woodland A.M. (2000). BRG1, a component of the SWI-SNF complex, is mutated in multiple human tumor cell lines. Cancer Res..

[45] Li J., Wang W., Zhang Y., Cieslik M., Guo J., Tan M., Green M.D., Wang W., Lin H., Li W. (2020). Epigenetic driver mutations in ARID1A shape cancer immune phenotype and immunotherapy. J. Clin. Investig..

[46] Cucchi D.G.J., Denys B., Kaspers G.J.L., Janssen J., Ossenkoppele G.J., de Haas V., Zwaan C.M., van den Heuvel-Eibrink M.M., Philippe J., Csikos T. (2018). RNA-based FLT3-ITD allelic ratio is associated with outcome and ex vivo response to FLT3 inhibitors in pediatric AML. Blood.

[47] Yu B.D., Hess J.L., Horning S.E., Brown G.A., Korsmeyer S.J. (1995). Altered Hox expression and segmental identity in Mll-mutant mice. Nature.

[48] Corral J., Lavenir I., Impey H., Warren A.J., Forster A., Larson T.A., Bell S., McKenzie A.N., King G., Rabbitts T.H. (1996). An Mll-AF9 fusion gene made by homologous recombination causes acute leukemia in chimeric mice: A method to create fusion oncogenes. Cell.

[49] Alawieh D., Cysique-Foinlan L., Willekens C., Renneville A. (2024). RAS mutations in myeloid malignancies: Revisiting old questions with novel insights and therapeutic perspectives. Blood Cancer J..

[50] Yang L., Rau R., Goodell M.A. (2015). DNMT3A in haematological malignancies. Nat. Rev. Cancer.

[51] Heyes E., Wilhelmson A.S., Wenzel A., Manhart G., Eder T., Schuster M.B., Rzepa E., Pundhir S., D’Altri T., Frank A.K. (2023). TET2 lesions enhance the aggressiveness of CEBPA-mutant acute myeloid leukemia by rebalancing GATA2 expression. Nat. Commun..

[52] Miyabayashi T., Teo J.L., Yamamoto M., McMillan M., Nguyen C., Kahn M. (2007). Wnt/β-catenin/CBP signaling maintains long-term murine embryonic stem cell pluripotency. Proc. Natl. Acad. Sci. USA.

[53] ten Berge D., Kurek D., Blauwkamp T., Koole W., Maas A., Eroglu E., Siu R.K., Nusse R. (2011). Embryonic stem cells require Wnt proteins to prevent differentiation to epiblast stem cells. Nat. Cell Biol..

[54] Marson A., Foreman R., Chevalier B., Bilodeau S., Kahn M., Young R.A., Jaenisch R. (2008). Wnt signaling promotes reprogramming of somatic cells to pluripotency. Cell Stem Cell.

[55] Zhou B., Lin W., Long Y., Yang Y., Zhang H., Wu K., Chu Q. (2022). Notch signaling pathway: Architecture, disease, and therapeutics. Signal Transduct. Target. Ther..

[56] Shi Q., Xue C., Zeng Y., Yuan X., Chu Q., Jiang S., Wang J., Zhang Y., Zhu D., Li L. (2024). Notch signaling pathway in cancer: From mechanistic insights to targeted therapies. Signal Transduct. Target. Ther..

[57] D’Assoro A.B., Leon-Ferre R., Braune E.B., Lendahl U. (2022). Roles of Notch Signaling in the Tumor Microenvironment. Int. J. Mol. Sci..

[58] Baroja I., Kyriakidis N.C., Halder G., Moya I.M. (2024). Expected and unexpected effects after systemic inhibition of Hippo transcriptional output in cancer. Nat. Commun..

[59] Ortega A., Vera I., Diaz M.P., Navarro C., Rojas M., Torres W., Parra H., Salazar J., De Sanctis J.B., Bermudez V. (2021). The YAP/TAZ Signaling Pathway in the Tumor Microenvironment and Carcinogenesis: Current Knowledge and Therapeutic Promises. Int. J. Mol. Sci..

[60] Sanchez-Vega F., Mina M., Armenia J., Chatila W.K., Luna A., La K.C., Dimitriadoy S., Liu D.L., Kantheti H.S., Saghafinia S. (2018). Oncogenic Signaling Pathways in The Cancer Genome Atlas. Cell.

[61] Yu F.X., Zhao B., Guan K.L. (2015). Hippo Pathway in Organ Size Control, Tissue Homeostasis, and Cancer. Cell.

[62] Laugesen A., Helin K. (2014). Chromatin repressive complexes in stem cells, development, and cancer. Cell Stem Cell.

[63] Romero P., Richart L., Aflaki S., Petitalot A., Burton M., Michaud A., Masliah-Planchon J., Kuhnowski F., Le Cam S., Balinas-Gavira C. (2024). EZH2 mutations in follicular lymphoma distort H3K27me3 profiles and alter transcriptional responses to PRC2 inhibition. Nat. Commun..

[64] Laugesen A., Hojfeldt J.W., Helin K. (2016). Role of the Polycomb Repressive Complex 2 (PRC2) in Transcriptional Regulation and Cancer. Cold Spring Harb. Perspect. Med..

[65] Beguelin W., Popovic R., Teater M., Jiang Y., Bunting K.L., Rosen M., Shen H., Yang S.N., Wang L., Ezponda T. (2013). EZH2 is required for germinal center formation and somatic EZH2 mutations promote lymphoid transformation. Cancer Cell.

[66] Dawson M.A., Kouzarides T. (2012). Cancer epigenetics: From mechanism to therapy. Cell.

[67] Kumar V.E., Nambiar R., De Souza C., Nguyen A., Chien J., Lam K.S. (2022). Targeting Epigenetic Modifiers of Tumor Plasticity and Cancer Stem Cell Behavior. Cells.

[68] Qi H., Pei D. (2007). The magic of four: Induction of pluripotent stem cells from somatic cells by Oct4, Sox2, Myc and Klf4. Cell Res..

[69] Takahashi K., Yamanaka S. (2006). Induction of pluripotent stem cells from mouse embryonic and adult fibroblast cultures by defined factors. Cell.

[70] Muller M., Hermann P.C., Liebau S., Weidgang C., Seufferlein T., Kleger A., Perkhofer L. (2016). The role of pluripotency factors to drive stemness in gastrointestinal cancer. Stem Cell Res..

[71] Dong Y., Tu R., Liu H., Qing G. (2020). Regulation of cancer cell metabolism: Oncogenic MYC in the driver’s seat. Signal Transduct. Target. Ther..

[72] Cazarin J., DeRollo R.E., Shahidan S., Burchett J.B., Mwangi D., Krishnaiah S., Hsieh A.L., Walton Z.E., Brooks R., Mello S.S. (2023). MYC disrupts transcriptional and metabolic circadian oscillations in cancer and promotes enhanced biosynthesis. PLoS Genet..

[73] Marques C., Unterkircher T., Kroon P., Oldrini B., Izzo A., Dramaretska Y., Ferrarese R., Kling E., Schnell O., Nelander S. (2021). NF1 regulates mesenchymal glioblastoma plasticity and aggressiveness through the AP-1 transcription factor FOSL1. eLife.

[74] Chen J., McKay R.M., Parada L.F. (2012). Malignant glioma: Lessons from genomics, mouse models, and stem cells. Cell.

[75] Liu F., Hon G.C., Villa G.R., Turner K.M., Ikegami S., Yang H., Ye Z., Li B., Kuan S., Lee A.Y. (2015). EGFR Mutation Promotes Glioblastoma through Epigenome and Transcription Factor Network Remodeling. Mol. Cell.

[76] Furnari F.B., Cloughesy T.F., Cavenee W.K., Mischel P.S. (2015). Heterogeneity of epidermal growth factor receptor signalling networks in glioblastoma. Nat. Rev. Cancer.

[77] White R.M., Zon L.I. (2008). Melanocytes in development, regeneration, and cancer. Cell Stem Cell.

[78] Uong A., Zon L.I. (2010). Melanocytes in development and cancer. J. Cell Physiol..

[79] Dave N., Guaita-Esteruelas S., Gutarra S., Frias A., Beltran M., Peiro S., de Herreros A.G. (2011). Functional cooperation between Snail1 and twist in the regulation of ZEB1 expression during epithelial to mesenchymal transition. J. Biol. Chem..

[80] Yang J., Antin P., Berx G., Blanpain C., Brabletz T., Bronner M., Campbell K., Cano A., Casanova J., Christofori G. (2020). Guidelines and definitions for research on epithelial-mesenchymal transition. Nat. Rev. Mol. Cell Biol..

[81] Dow L.E., O’Rourke K.P., Simon J., Tschaharganeh D.F., van Es J.H., Clevers H., Lowe S.W. (2015). Apc Restoration Promotes Cellular Differentiation and Reestablishes Crypt Homeostasis in Colorectal Cancer. Cell.

[82] Schwitalla S., Fingerle A.A., Cammareri P., Nebelsiek T., Goktuna S.I., Ziegler P.K., Canli O., Heijmans J., Huels D.J., Moreaux G. (2013). Intestinal tumorigenesis initiated by dedifferentiation and acquisition of stem-cell-like properties. Cell.

[83] Verhagen M.P., Joosten R., Schmitt M., Valimaki N., Sacchetti A., Rajamaki K., Choi J., Procopio P., Silva S., van der Steen B. (2024). Non-stem cell lineages as an alternative origin of intestinal tumorigenesis in the context of inflammation. Nat. Genet..

[84] Storz P. (2017). Acinar cell plasticity and development of pancreatic ductal adenocarcinoma. Nat. Rev. Gastroenterol. Hepatol..

[85] Kalluri R., Weinberg R.A. (2009). The basics of epithelial-mesenchymal transition. J. Clin. Investig..

[86] Lamouille S., Xu J., Derynck R. (2014). Molecular mechanisms of epithelial-mesenchymal transition. Nat. Rev. Mol. Cell Biol..

[87] Fontana R., Mestre-Farrera A., Yang J. (2024). Update on Epithelial-Mesenchymal Plasticity in Cancer Progression. Annu. Rev. Pathol. Mech. Dis..

[88] Feigin M.E., Muthuswamy S.K. (2009). Polarity proteins regulate mammalian cell-cell junctions and cancer pathogenesis. Curr. Opin. Cell Biol..

[89] Coradini D., Casarsa C., Oriana S. (2011). Epithelial cell polarity and tumorigenesis: New perspectives for cancer detection and treatment. Acta Pharmacol. Sin..

[90] Kaufhold S., Bonavida B. (2014). Central role of Snail1 in the regulation of EMT and resistance in cancer: A target for therapeutic intervention. J. Exp. Clin. Cancer Res..

[91] Wang G., Guo X., Hong W., Liu Q., Wei T., Lu C., Gao L., Ye D., Zhou Y., Chen J. (2013). Critical regulation of miR-200/ZEB2 pathway in Oct4/Sox2-induced mesenchymal-to-epithelial transition and induced pluripotent stem cell generation. Proc. Natl. Acad. Sci. USA.

[92] Saxena M., Stephens M.A., Pathak H., Rangarajan A. (2011). Transcription factors that mediate epithelial-mesenchymal transition lead to multidrug resistance by upregulating ABC transporters. Cell Death Dis..

[93] Xue W., Yang L., Chen C., Ashrafizadeh M., Tian Y., Sun R. (2024). Wnt/β-catenin-driven EMT regulation in human cancers. Cell Mol. Life Sci..

[94] Taipale J., Beachy P.A. (2001). The Hedgehog and Wnt signalling pathways in cancer. Nature.

[95] Begicevic R.R., Falasca M. (2017). ABC Transporters in Cancer Stem Cells: Beyond Chemoresistance. Int. J. Mol. Sci..

[96] Jinesh G.G., Brohl A.S. (2022). Classical epithelial-mesenchymal transition (EMT) and alternative cell death process-driven blebbishield metastatic-witch (BMW) pathways to cancer metastasis. Signal Transduct. Target. Ther..

[97] Wang G., Xu D., Zhang Z., Li X., Shi J., Sun J., Liu H.Z., Li X., Zhou M., Zheng T. (2021). The pan-cancer landscape of crosstalk between epithelial-mesenchymal transition and immune evasion relevant to prognosis and immunotherapy response. NPJ Precis. Oncol..

[98] Zhou H.M., Zhang J.G., Zhang X., Li Q. (2021). Targeting cancer stem cells for reversing therapy resistance: Mechanism, signaling, and prospective agents. Signal Transduct. Target. Ther..

[99] Wu H.T., Zhong H.T., Li G.W., Shen J.X., Ye Q.Q., Zhang M.L., Liu J. (2020). Oncogenic functions of the EMT-related transcription factor ZEB1 in breast cancer. J. Transl. Med..

[100] Zhao H., Ming T., Tang S., Ren S., Yang H., Liu M., Tao Q., Xu H. (2022). Wnt signaling in colorectal cancer: Pathogenic role and therapeutic target. Mol. Cancer.

[101] Shi X., Yang J., Deng S., Xu H., Wu D., Zeng Q., Wang S., Hu T., Wu F., Zhou H. (2022). TGF-beta signaling in the tumor metabolic microenvironment and targeted therapies. J. Hematol. Oncol..

[102] Chen Z., Han F., Du Y., Shi H., Zhou W. (2023). Hypoxic microenvironment in cancer: Molecular mechanisms and therapeutic interventions. Signal Transduct. Target. Ther..

[103] Kim B.N., Ahn D.H., Kang N., Yeo C.D., Kim Y.K., Lee K.Y., Kim T.J., Lee S.H., Park M.S., Yim H.W. (2020). TGF-beta induced EMT and stemness characteristics are associated with epigenetic regulation in lung cancer. Sci. Rep..

[104] Dardare J., Witz A., Merlin J.L., Bochnakian A., Toussaint P., Gilson P., Harle A. (2021). Epithelial to Mesenchymal Transition in Patients with Pancreatic Ductal Adenocarcinoma: State-of-the-Art and Therapeutic Opportunities. Pharmaceuticals.

[105] Wang S., Zheng Y., Yang F., Zhu L., Zhu X.Q., Wang Z.F., Wu X.L., Zhou C.H., Yan J.Y., Hu B.Y. (2021). The molecular biology of pancreatic adenocarcinoma: Translational challenges and clinical perspectives. Signal Transduct. Target. Ther..

[106] Dai J., Su Y., Zhong S., Cong L., Liu B., Yang J., Tao Y., He Z., Chen C., Jiang Y. (2020). Exosomes: Key players in cancer and potential therapeutic strategy. Signal Transduct. Target. Ther..

[107] Scioli M.G., Terriaca S., Fiorelli E., Storti G., Fabbri G., Cervelli V., Orlandi A. (2021). Extracellular Vesicles and Cancer Stem Cells in Tumor Progression: New Therapeutic Perspectives. Int. J. Mol. Sci..

[108] Su C., Zhang J., Yarden Y., Fu L. (2021). The key roles of cancer stem cell-derived extracellular vesicles. Signal Transduct. Target. Ther..

[109] Zeijlemaker W., Grob T., Meijer R., Hanekamp D., Kelder A., Carbaat-Ham J.C., Oussoren-Brockhoff Y.J.M., Snel A.N., Veldhuizen D., Scholten W.J. (2019). CD34^+^CD38^−^ leukemic stem cell frequency to predict outcome in acute myeloid leukemia. Leukemia.

[110] Gerber J.M., Smith B.D., Ngwang B., Zhang H., Vala M.S., Morsberger L., Galkin S., Collector M.I., Perkins B., Levis M.J. (2012). A clinically relevant population of leukemic CD34^+^CD38^−^ cells in acute myeloid leukemia. Blood.

[111] Glumac P.M., LeBeau A.M. (2018). The role of CD133 in cancer: A concise review. Clin. Transl. Med..

[112] Smith L.M., Nesterova A., Ryan M.C., Duniho S., Jonas M., Anderson M., Zabinski R.F., Sutherland M.K., Gerber H.P., Van Orden K.L. (2008). CD133/prominin-1 is a potential therapeutic target for antibody-drug conjugates in hepatocellular and gastric cancers. Br. J. Cancer.

[113] Tang X., Zuo C., Fang P., Liu G., Qiu Y., Huang Y., Tang R. (2021). Targeting Glioblastoma Stem Cells: A Review on Biomarkers, Signal Pathways and Targeted Therapy. Front. Oncol..

[114] Xu X., Chai S., Wang P., Zhang C., Yang Y., Yang Y., Wang K. (2015). Aldehyde dehydrogenases and cancer stem cells. Cancer Lett..

[115] Wei Y., Li Y., Chen Y., Liu P., Huang S., Zhang Y., Sun Y., Wu Z., Hu M., Wu Q. (2022). ALDH1: A potential therapeutic target for cancer stem cells in solid tumors. Front. Oncol..

[116] Yamashita T., Budhu A., Forgues M., Wang X.W. (2007). Activation of hepatic stem cell marker EpCAM by Wnt-β-catenin signaling in hepatocellular carcinoma. Cancer Res..

[117] Gires O., Klein C.A., Baeuerle P.A. (2009). On the abundance of EpCAM on cancer stem cells. Nat. Rev. Cancer.

[118] Baeuerle P.A., Gires O. (2007). EpCAM (CD326) finding its role in cancer. Br. J. Cancer.

[119] Jin X., Jin X., Jung J.E., Beck S., Kim H. (2013). Cell surface Nestin is a biomarker for glioma stem cells. Biochem. Biophys. Res. Commun..

[120] Qiao X., Zhang Y., Sun L., Ma Q., Yang J., Ai L., Xue J., Chen G., Zhang H., Ji C. (2021). Association of human breast cancer CD44(-)/CD24(-) cells with delayed distant metastasis. eLife.

[121] Bahmad H.F., Cheaito K., Chalhoub R.M., Hadadeh O., Monzer A., Ballout F., El-Hajj A., Mukherji D., Liu Y.N., Daoud G. (2018). Sphere-Formation Assay: Three-Dimensional in vitro Culturing of Prostate Cancer Stem/Progenitor Sphere-Forming Cells. Front. Oncol..

[122] Zhou J., Wang C.Y., Liu T., Wu B., Zhou F., Xiong J.X., Wu H.S., Tao J., Zhao G., Yang M. (2008). Persistence of side population cells with high drug efflux capacity in pancreatic cancer. World J. Gastroenterol..

[123] Del Vecchio V., La Noce M., Tirino V. (2024). ALDH Activity Assay: A Method for Cancer Stem Cell (CSC) Identification and Isolation. Methods Mol. Biol..

[124] Kirstetter P., Anderson K., Porse B.T., Jacobsen S.E., Nerlov C. (2006). Activation of the canonical Wnt pathway leads to loss of hematopoietic stem cell repopulation and multilineage differentiation block. Nat. Immunol..

[125] Klaus A., Birchmeier W. (2008). Wnt signalling and its impact on development and cancer. Nat. Rev. Cancer.

[126] Takebe N., Miele L., Harris P.J., Jeong W., Bando H., Kahn M., Yang S.X., Ivy S.P. (2015). Targeting Notch, Hedgehog, and Wnt pathways in cancer stem cells: Clinical update. Nat. Rev. Clin. Oncol..

[127] Kumar V., Vashishta M., Kong L., Wu X., Lu J.J., Guha C., Dwarakanath B.S. (2021). The Role of Notch, Hedgehog, and Wnt Signaling Pathways in the Resistance of Tumors to Anticancer Therapies. Front. Cell Dev. Biol..

[128] Yang L., Shi P., Zhao G., Xu J., Peng W., Zhang J., Zhang G., Wang X., Dong Z., Chen F. (2020). Targeting cancer stem cell pathways for cancer therapy. Signal Transduct. Target. Ther..

[129] Holland J.D., Klaus A., Garratt A.N., Birchmeier W. (2013). Wnt signaling in stem and cancer stem cells. Curr. Opin. Cell Biol..

[130] Jun S., Jung Y.S., Suh H.N., Wang W., Kim M.J., Oh Y.S., Lien E.M., Shen X., Matsumoto Y., McCrea P.D. (2016). LIG4 mediates Wnt signalling-induced radioresistance. Nat. Commun..

[131] Tao S., Tang D., Morita Y., Sperka T., Omrani O., Lechel A., Sakk V., Kraus J., Kestler H.A., Kuhl M. (2017). Wnt activity and basal niche position sensitize intestinal stem and progenitor cells to DNA damage. EMBO J..

[132] Chen S., Guttridge D.C., You Z., Zhang Z., Fribley A., Mayo M.W., Kitajewski J., Wang C.Y. (2001). Wnt-1 signaling inhibits apoptosis by activating β-catenin/T cell factor-mediated transcription. J. Cell Biol..

[133] Correa S., Binato R., Du Rocher B., Castelo-Branco M.T., Pizzatti L., Abdelhay E. (2012). Wnt/β-catenin pathway regulates ABCB1 transcription in chronic myeloid leukemia. BMC Cancer.

[134] Stein U., Fleuter C., Siegel F., Smith J., Kopacek A., Scudiero D.A., Hite K.M., Schlag P.M., Shoemaker R.H., Walther W. (2012). Impact of mutant beta-catenin on ABCB1 expression and therapy response in colon cancer cells. Br. J. Cancer.

[135] Zhao F., Xiao C., Evans K.S., Theivanthiran T., DeVito N., Holtzhausen A., Liu J., Liu X., Boczkowski D., Nair S. (2018). Paracrine Wnt5a-beta-Catenin Signaling Triggers a Metabolic Program that Drives Dendritic Cell Tolerization. Immunity.

[136] Suryawanshi A., Hussein M.S., Prasad P.D., Manicassamy S. (2020). Wnt Signaling Cascade in Dendritic Cells and Regulation of Anti-tumor Immunity. Front. Immunol..

[137] Li X., Xiang Y., Li F., Yin C., Li B., Ke X. (2019). WNT/β-Catenin Signaling Pathway Regulating T Cell-Inflammation in the Tumor Microenvironment. Front. Immunol..

[138] Yu F., Yu C., Li F., Zuo Y., Wang Y., Yao L., Wu C., Wang C., Ye L. (2021). Wnt/β-catenin signaling in cancers and targeted therapies. Signal Transduct. Target. Ther..

[139] Jing J., Wu Z., Wang J., Luo G., Lin H., Fan Y., Zhou C. (2023). Hedgehog signaling in tissue homeostasis, cancers, and targeted therapies. Signal Transduct. Target. Ther..

[140] Wang C.Y., Chang Y.C., Kuo Y.L., Lee K.T., Chen P.S., Cheung C.H.A., Chang C.P., Phan N.N., Shen M.R., Hsu H.P. (2019). Mutation of the PTCH1 gene predicts recurrence of breast cancer. Sci. Rep..

[141] Faiao-Flores F., Alves-Fernandes D.K., Pennacchi P.C., Sandri S., Vicente A.L., Scapulatempo-Neto C., Vazquez V.L., Reis R.M., Chauhan J., Goding C.R. (2017). Targeting the hedgehog transcription factors GLI1 and GLI2 restores sensitivity to vemurafenib-resistant human melanoma cells. Oncogene.

[142] Chai J.Y., Sugumar V., Alshanon A.F., Wong W.F., Fung S.Y., Looi C.Y. (2021). Defining the Role of GLI/Hedgehog Signaling in Chemoresistance: Implications in Therapeutic Approaches. Cancers.

[143] Po A., Citarella A., Catanzaro G., Besharat Z.M., Trocchianesi S., Gianno F., Sabato C., Moretti M., De Smaele E., Vacca A. (2020). Hedgehog-GLI signalling promotes chemoresistance through the regulation of ABC transporters in colorectal cancer cells. Sci. Rep..

[144] Meng E., Hanna A., Samant R.S., Shevde L.A. (2015). The Impact of Hedgehog Signaling Pathway on DNA Repair Mechanisms in Human Cancer. Cancers.

[145] Kanda S., Mitsuyasu T., Nakao Y., Kawano S., Goto Y., Matsubara R., Nakamura S. (2013). Anti-apoptotic role of the sonic hedgehog signaling pathway in the proliferation of ameloblastoma. Int. J. Oncol..

[146] Wang F., Ma L., Zhang Z., Liu X., Gao H., Zhuang Y., Yang P., Kornmann M., Tian X., Yang Y. (2016). Hedgehog Signaling Regulates Epithelial-Mesenchymal Transition in Pancreatic Cancer Stem-Like Cells. J. Cancer.

[147] Ohta H., Aoyagi K., Fukaya M., Danjoh I., Ohta A., Isohata N., Saeki N., Taniguchi H., Sakamoto H., Shimoda T. (2009). Cross talk between hedgehog and epithelial-mesenchymal transition pathways in gastric pit cells and in diffuse-type gastric cancers. Br. J. Cancer.

[148] Wang J., Cui B., Li X., Zhao X., Huang T., Ding X. (2023). The emerging roles of Hedgehog signaling in tumor immune microenvironment. Front. Oncol..

[149] Giammona A., Crivaro E., Stecca B. (2023). Emerging Roles of Hedgehog Signaling in Cancer Immunity. Int. J. Mol. Sci..

[150] Wang M., Yu F., Zhang Y., Li P. (2024). Novel insights into Notch signaling in tumor immunity: Potential targets for cancer immunotherapy. Front. Immunol..

[151] Glaviano A., Foo A.S.C., Lam H.Y., Yap K.C.H., Jacot W., Jones R.H., Eng H., Nair M.G., Makvandi P., Geoerger B. (2023). PI3K/AKT/mTOR signaling transduction pathway and targeted therapies in cancer. Mol. Cancer.

[152] Peng Y., Wang Y., Zhou C., Mei W., Zeng C. (2022). PI3K/Akt/mTOR Pathway and Its Role in Cancer Therapeutics: Are We Making Headway?. Front. Oncol..

[153] He Y., Sun M.M., Zhang G.G., Yang J., Chen K.S., Xu W.W., Li B. (2021). Targeting PI3K/Akt signal transduction for cancer therapy. Signal Transduct. Target. Ther..

[154] Son B., Lee W., Kim H., Shin H., Park H.H. (2024). Targeted therapy of cancer stem cells: Inhibition of mTOR in pre-clinical and clinical research. Cell Death Dis..

[155] Karami Fath M., Ebrahimi M., Nourbakhsh E., Zia Hazara A., Mirzaei A., Shafieyari S., Salehi A., Hoseinzadeh M., Payandeh Z., Barati G. (2022). PI3K/Akt/mTOR signaling pathway in cancer stem cells. Pathol. Res. Pract..

[156] Xia P., Xu X.Y. (2015). PI3K/Akt/mTOR signaling pathway in cancer stem cells: From basic research to clinical application. Am. J. Cancer Res..

[157] Heddleston J.M., Li Z., Lathia J.D., Bao S., Hjelmeland A.B., Rich J.N. (2010). Hypoxia inducible factors in cancer stem cells. Br. J. Cancer.

[158] Keith B., Simon M.C. (2007). Hypoxia-inducible factors, stem cells, and cancer. Cell.

[159] Tong W.W., Tong G.H., Liu Y. (2018). Cancer stem cells and hypoxia-inducible factors (Review). Int. J. Oncol..

[160] Thews O., Gassner B., Kelleher D.K., Gekle M. (2008). Activity of drug efflux transporters in tumor cells under hypoxic conditions. Adv. Exp. Med. Biol..

[161] Tafech A., Stephanou A. (2024). On the Importance of Acidity in Cancer Cells and Therapy. Biology.

[162] Vander Linden C., Corbet C. (2019). Therapeutic Targeting of Cancer Stem Cells: Integrating and Exploiting the Acidic Niche. Front. Oncol..

[163] Rolver M.G., Camacho-Roda J., Dai Y., Flinck M., Ialchina R., Hindkaer J., Dyhr R.T., Bodilsen A.N., Prasad N.S., Baldan J. (2025). Tumor microenvironment acidosis favors pancreatic cancer stem cell properties and in vivo metastasis. iScience.

[164] Cardone R.A., Casavola V., Reshkin S.J. (2005). The role of disturbed pH dynamics and the Na^+^/H^+^ exchanger in metastasis. Nat. Rev. Cancer.

[165] Harguindey S., Orive G., Luis Pedraz J., Paradiso A., Reshkin S.J. (2005). The role of pH dynamics and the Na^+^/H^+^ antiporter in the etiopathogenesis and treatment of cancer. Two faces of the same coin—One single nature. Biochim. Biophys. Acta.

[166] Peppicelli S., Andreucci E., Ruzzolini J., Laurenzana A., Margheri F., Fibbi G., Del Rosso M., Bianchini F., Calorini L. (2017). The acidic microenvironment as a possible niche of dormant tumor cells. Cell Mol. Life Sci..

[167] Cheng G.M., To K.K. (2012). Adverse Cell Culture Conditions Mimicking the Tumor Microenvironment Upregulate ABCG2 to Mediate Multidrug Resistance and a More Malignant Phenotype. ISRN Oncol..

[168] Visioli F., Wang Y., Alam G.N., Ning Y., Rados P.V., Nor J.E., Polverini P.J. (2014). Glucose-regulated protein 78 (Grp78) confers chemoresistance to tumor endothelial cells under acidic stress. PLoS ONE.

[169] Thews O., Nowak M., Sauvant C., Gekle M. (2011). Hypoxia-induced extracellular acidosis increases p-glycoprotein activity and chemoresistance in tumors in vivo via p38 signaling pathway. Adv. Exp. Med. Biol..

[170] Sharma P., Allison J.P. (2015). The future of immune checkpoint therapy. Science.

[171] Mahoney K.M., Rennert P.D., Freeman G.J. (2015). Combination cancer immunotherapy and new immunomodulatory targets. Nat. Rev. Drug Discov..

[172] Pardoll D.M. (2012). The blockade of immune checkpoints in cancer immunotherapy. Nat. Rev. Cancer.

[173] Galassi C., Musella M., Manduca N., Maccafeo E., Sistigu A. (2021). The Immune Privilege of Cancer Stem Cells: A Key to Understanding Tumor Immune Escape and Therapy Failure. Cells.

[174] Li Y.R., Fang Y., Lyu Z., Zhu Y., Yang L. (2023). Exploring the dynamic interplay between cancer stem cells and the tumor microenvironment: Implications for novel therapeutic strategies. J. Transl. Med..

[175] Chen P., Hsu W.H., Han J., Xia Y., DePinho R.A. (2021). Cancer Stemness Meets Immunity: From Mechanism to Therapy. Cell Rep..

[176] Vito A., El-Sayes N., Mossman K. (2020). Hypoxia-Driven Immune Escape in the Tumor Microenvironment. Cells.

[177] Guo T., Xu J. (2024). Cancer-associated fibroblasts: A versatile mediator in tumor progression, metastasis, and targeted therapy. Cancer Metastasis Rev..

[178] Wu F., Yang J., Liu J., Wang Y., Mu J., Zeng Q., Deng S., Zhou H. (2021). Signaling pathways in cancer-associated fibroblasts and targeted therapy for cancer. Signal Transduct. Target. Ther..

[179] Prager B.C., Xie Q., Bao S., Rich J.N. (2019). Cancer Stem Cells: The Architects of the Tumor Ecosystem. Cell Stem Cell.

[180] Lathia J.D., Heddleston J.M., Venere M., Rich J.N. (2011). Deadly teamwork: Neural cancer stem cells and the tumor microenvironment. Cell Stem Cell.

[181] Plaks V., Kong N., Werb Z. (2015). The cancer stem cell niche: How essential is the niche in regulating stemness of tumor cells?. Cell Stem Cell.

[182] Cabarcas S.M., Mathews L.A., Farrar W.L. (2011). The cancer stem cell niche--there goes the neighborhood?. Int. J. Cancer.

[183] Liu X., Ye Y., Zhu L., Xiao X., Zhou B., Gu Y., Si H., Liang H., Liu M., Li J. (2023). Niche stiffness sustains cancer stemness via TAZ and NANOG phase separation. Nat. Commun..

[184] Dzobo K., Dandara C. (2023). The Extracellular Matrix: Its Composition, Function, Remodeling, and Role in Tumorigenesis. Biomimetics.

[185] Chen J.R., Zhao J.T., Xie Z.Z. (2022). Integrin-mediated cancer progression as a specific target in clinical therapy. Biomed. Pharmacother..

[186] Cooper J., Giancotti F.G. (2019). Integrin Signaling in Cancer: Mechanotransduction, Stemness, Epithelial Plasticity, and Therapeutic Resistance. Cancer Cell.

[187] Nonnast E., Mira E., Manes S. (2025). The role of laminins in cancer pathobiology: A comprehensive review. J. Transl. Med..

[188] Givant-Horwitz V., Davidson B., Reich R. (2005). Laminin-induced signaling in tumor cells. Cancer Lett..

[189] Lokeshwar V.B., Mirza S., Jordan A. (2014). Targeting hyaluronic acid family for cancer chemoprevention and therapy. Adv. Cancer Res..

[190] Bhattacharyya M., Jariyal H., Srivastava A. (2023). Hyaluronic acid: More than a carrier, having an overpowering extracellular and intracellular impact on cancer. Carbohydr. Polym..

[191] Michalczyk M., Humeniuk E., Adamczuk G., Korga-Plewko A. (2022). Hyaluronic Acid as a Modern Approach in Anticancer Therapy-Review. Int. J. Mol. Sci..

[192] Lambert A.W., Wong C.K., Ozturk S., Papageorgis P., Raghunathan R., Alekseyev Y., Gower A.C., Reinhard B.M., Abdolmaleky H.M., Thiagalingam S. (2016). Tumor Cell-Derived Periostin Regulates Cytokines That Maintain Breast Cancer Stem Cells. Mol. Cancer Res..

[193] Dorafshan S., Razmi M., Safaei S., Gentilin E., Madjd Z., Ghods R. (2022). Periostin: Biology and function in cancer. Cancer Cell Int..

[194] Gonzalez-Gonzalez L., Alonso J. (2018). Periostin: A Matricellular Protein With Multiple Functions in Cancer Development and Progression. Front. Oncol..

[195] Seno M. (2025). A Landscape of Cancer Initiation and Cancer Stem Cells. Cancers.

[196] Leck L.Y.W., Abd El-Aziz Y.S., McKelvey K.J., Park K.C., Sahni S., Lane D.J.R., Skoda J., Jansson P.J. (2024). Cancer stem cells: Masters of all traits. Biochim. Biophys. Acta Mol. Basis Dis..

[197] O’Brien C.A., Kreso A., Jamieson C.H. (2010). Cancer stem cells and self-renewal. Clin. Cancer Res..

[198] Rich J.N. (2016). Cancer stem cells: Understanding tumor hierarchy and heterogeneity. Medicine.

[199] Lionetti M.C., Fumagalli M.R., La Porta C.A.M. (2020). Cancer stem cells, plasticity, and drug resistance. Cancer Drug Resist..

[200] Nandy S.B., Lakshmanaswamy R. (2017). Cancer Stem Cells and Metastasis. Prog. Mol. Biol. Transl. Sci..

[201] Chowdhury F., Huang B., Wang N. (2022). Forces in stem cells and cancer stem cells. Cells Dev..

[202] Liu N., Li S., Wu N., Cho K.S. (2017). Acetylation and deacetylation in cancer stem-like cells. Oncotarget.

[203] Wainwright E.N., Scaffidi P. (2017). Epigenetics and Cancer Stem Cells: Unleashing, Hijacking, and Restricting Cellular Plasticity. Trends Cancer.

[204] Verona F., Pantina V.D., Modica C., Lo Iacono M., D’Accardo C., Porcelli G., Cricchio D., Turdo A., Gaggianesi M., Di Franco S. (2022). Targeting epigenetic alterations in cancer stem cells. Front. Mol. Med..

[205] Esteller M., Dawson M.A., Kadoch C., Rassool F.V., Jones P.A., Baylin S.B. (2024). The Epigenetic Hallmarks of Cancer. Cancer Discov..

[206] Tang D.G. (2012). Understanding cancer stem cell heterogeneity and plasticity. Cell Res..

[207] Eun K., Ham S.W., Kim H. (2017). Cancer stem cell heterogeneity: Origin and new perspectives on CSC targeting. BMB Rep..

[208] Pietras A. (2011). Cancer stem cells in tumor heterogeneity. Adv. Cancer Res..

[209] Bautch V.L. (2010). Cancer: Tumour stem cells switch sides. Nature.

[210] Malanchi I., Santamaria-Martinez A., Susanto E., Peng H., Lehr H.A., Delaloye J.F., Huelsken J. (2011). Interactions between cancer stem cells and their niche govern metastatic colonization. Nature.

[211] Bjerkvig R., Johansson M., Miletic H., Niclou S.P. (2009). Cancer stem cells and angiogenesis. Semin. Cancer Biol..

[212] Flores D.G., Ledur P.F., Abujamra A.L., Brunetto A.L., Schwartsmann G., Lenz G., Roesler R. (2009). Cancer stem cells and the biology of brain tumors. Curr. Stem Cell Res. Ther..

[213] Piccirillo S.G., Binda E., Fiocco R., Vescovi A.L., Shah K. (2009). Brain cancer stem cells. J. Mol. Med..

[214] Denysenko T., Gennero L., Roos M.A., Melcarne A., Juenemann C., Faccani G., Morra I., Cavallo G., Reguzzi S., Pescarmona G. (2010). Glioblastoma cancer stem cells: Heterogeneity, microenvironment and related therapeutic strategies. Cell Biochem. Funct..

[215] Ohlund D., Elyada E., Tuveson D. (2014). Fibroblast heterogeneity in the cancer wound. J. Exp. Med..

[216] Qin X., Guo H., Wang X., Zhu X., Yan M., Wang X., Xu Q., Shi J., Lu E., Chen W. (2019). Exosomal miR-196a derived from cancer-associated fibroblasts confers cisplatin resistance in head and neck cancer through targeting CDKN1B and ING5. Genome Biol..

[217] Qin X., Yan M., Zhang J., Wang X., Shen Z., Lv Z., Li Z., Wei W., Chen W. (2016). TGFβ3-mediated induction of Periostin facilitates head and neck cancer growth and is associated with metastasis. Sci. Rep..

[218] Alvarez-Teijeiro S., Garcia-Inclan C., Villaronga M.A., Casado P., Hermida-Prado F., Granda-Diaz R., Rodrigo J.P., Calvo F., Del-Rio-Ibisate N., Gandarillas A. (2018). Factors Secreted by Cancer-Associated Fibroblasts that Sustain Cancer Stem Properties in Head and Neck Squamous Carcinoma Cells as Potential Therapeutic Targets. Cancers.

[219] Jiang J., Ye F., Yang X., Zong C., Gao L., Yang Y., Zhao Q., Han Z., Wei L. (2017). Peri-tumor associated fibroblasts promote intrahepatic metastasis of hepatocellular carcinoma by recruiting cancer stem cells. Cancer Lett..

[220] Wang S.S., Gao X.L., Liu X., Gao S.Y., Fan Y.L., Jiang Y.P., Ma X.R., Jiang J., Feng H., Chen Q.M. (2016). CD133+ cancer stem-like cells promote migration and invasion of salivary adenoid cystic carcinoma by inducing vasculogenic mimicry formation. Oncotarget.

[221] Murai T., Matsuda S. (2023). Targeting the PI3K-Akt-mTOR signaling pathway involved in vasculogenic mimicry promoted by cancer stem cells. Am. J. Cancer Res..

[222] Lizarraga-Verdugo E., Avendano-Felix M., Bermudez M., Ramos-Payan R., Perez-Plasencia C., Aguilar-Medina M. (2020). Cancer Stem Cells and Its Role in Angiogenesis and Vasculogenic Mimicry in Gastrointestinal Cancers. Front. Oncol..

[223] Lin D., Shen L., Luo M., Zhang K., Li J., Yang Q., Zhu F., Zhou D., Zheng S., Chen Y. (2021). Circulating tumor cells: Biology and clinical significance. Signal Transduct. Target. Ther..

[224] Gu X., Wei S., Lv X. (2024). Circulating tumor cells: From new biological insights to clinical practice. Signal Transduct. Target. Ther..

[225] Aramini B., Masciale V., Arienti C., Dominici M., Stella F., Martinelli G., Fabbri F. (2022). Cancer Stem Cells (CSCs), Circulating Tumor Cells (CTCs) and Their Interplay with Cancer Associated Fibroblasts (CAFs): A New World of Targets and Treatments. Cancers.

[226] Li W., Ma H., Zhang J., Zhu L., Wang C., Yang Y. (2017). Unraveling the roles of CD44/CD24 and ALDH1 as cancer stem cell markers in tumorigenesis and metastasis. Sci. Rep..

[227] Xu H., Niu M., Yuan X., Wu K., Liu A. (2020). CD44 as a tumor biomarker and therapeutic target. Exp. Hematol. Oncol..

[228] Li D., Guo X., Yang K., Yang Y., Zhou W., Huang Y., Liang X., Su J., Jiang L., Li J. (2023). EpCAM-targeting CAR-T cell immunotherapy is safe and efficacious for epithelial tumors. Sci. Adv..

[229] Zeng S., Jin N., Yu B., Ren Q., Yan Z., Fu S. (2024). Chimeric antigen receptor-T cells targeting epithelial cell adhesion molecule antigens are effective in the treatment of colorectal cancer. BMC Gastroenterol..

[230] Ooki A., VandenBussche C.J., Kates M., Hahn N.M., Matoso A., McConkey D.J., Bivalacqua T.J., Hoque M.O. (2018). CD24 regulates cancer stem cell (CSC)-like traits and a panel of CSC-related molecules serves as a non-invasive urinary biomarker for the detection of bladder cancer. Br. J. Cancer.

[231] Jang Y., Kang S., Han H.H., Kim B.G., Cho N.H. (2024). CD24 induced cellular quiescence-like state and chemoresistance in ovarian cancer cells via miR-130a/301a-dependent CDK19 downregulation. Cell Death Discov..

[232] Yang Y., Zhu G., Yang L., Yang Y. (2023). Targeting CD24 as a novel immunotherapy for solid cancers. Cell Commun. Signal.

[233] Ran Y., Hossain F., Pannuti A., Lessard C.B., Ladd G.Z., Jung J.I., Minter L.M., Osborne B.A., Miele L., Golde T.E. (2017). gamma-Secretase inhibitors in cancer clinical trials are pharmacologically and functionally distinct. EMBO Mol. Med..

[234] Feng M., Santhanam R.K., Xing H., Zhou M., Jia H. (2024). Inhibition of γ-secretase/Notch pathway as a potential therapy for reversing cancer drug resistance. Biochem. Pharmacol..

[235] Slack R.J., Macdonald S.J.F., Roper J.A., Jenkins R.G., Hatley R.J.D. (2022). Emerging therapeutic opportunities for integrin inhibitors. Nat. Rev. Drug Discov..

[236] Chen H.C., Mueller N., Stott K., Kapeni C., Rivers E., Sauer C.M., Beke F., Walsh S.J., Ashman N., O’Brien L. (2024). Novel immunotherapeutics against LGR5 to target multiple cancer types. EMBO Mol. Med..

[237] Zhou M., Liu C., Li B., Li J., Zhang P., Huang Y., Li L. (2024). Cell surface patching via CXCR4-targeted nanothreads for cancer metastasis inhibition. Nat. Commun..

[238] Leo M., Sabatino L. (2022). Targeting CXCR4 and CD47 Receptors: An Overview of New and Old Molecules for a Biological Personalized Anticancer Therapy. Int. J. Mol. Sci..

[239] Takahashi-Yanaga F., Kahn M. (2010). Targeting Wnt signaling: Can we safely eradicate cancer stem cells?. Clin. Cancer Res..

[240] Wend P., Holland J.D., Ziebold U., Birchmeier W. (2010). Wnt signaling in stem and cancer stem cells. Semin. Cell Dev. Biol..

[241] Liu Y., Qi X., Donnelly L., Elghobashi-Meinhardt N., Long T., Zhou R.W., Sun Y., Wang B., Li X. (2022). Mechanisms and inhibition of Porcupine-mediated Wnt acylation. Nature.

[242] Shah K., Panchal S., Patel B. (2021). Porcupine inhibitors: Novel and emerging anti-cancer therapeutics targeting the Wnt signaling pathway. Pharmacol. Res..

[243] Liu J., Pan S., Hsieh M.H., Ng N., Sun F., Wang T., Kasibhatla S., Schuller A.G., Li A.G., Cheng D. (2013). Targeting Wnt-driven cancer through the inhibition of Porcupine by LGK974. Proc. Natl. Acad. Sci. USA.

[244] Huang S.M., Mishina Y.M., Liu S., Cheung A., Stegmeier F., Michaud G.A., Charlat O., Wiellette E., Zhang Y., Wiessner S. (2009). Tankyrase inhibition stabilizes axin and antagonizes Wnt signalling. Nature.

[245] Liu J., Xiao Q., Xiao J., Niu C., Li Y., Zhang X., Zhou Z., Shu G., Yin G. (2022). Wnt/β-catenin signalling: Function, biological mechanisms, and therapeutic opportunities. Signal Transduct. Target. Ther..

[246] Hirakawa T., Nasu K., Miyabe S., Kouji H., Katoh A., Uemura N., Narahara H. (2019). β-catenin signaling inhibitors ICG-001 and C-82 improve fibrosis in preclinical models of endometriosis. Sci. Rep..

[247] Lin H.H., Feng W.C., Lu L.C., Shao Y.Y., Hsu C.H., Cheng A.L. (2016). Inhibition of the Wnt/β-catenin signaling pathway improves the anti-tumor effects of sorafenib against hepatocellular carcinoma. Cancer Lett..

[248] DeVito N.C., Sturdivant M., Thievanthiran B., Xiao C., Plebanek M.P., Salama A.K.S., Beasley G.M., Holtzhausen A., Novotny-Diermayr V., Strickler J.H. (2021). Pharmacological Wnt ligand inhibition overcomes key tumor-mediated resistance pathways to anti-PD-1 immunotherapy. Cell Rep..

[249] Diamond J.R., Becerra C., Richards D., Mita A., Osborne C., O’Shaughnessy J., Zhang C., Henner R., Kapoun A.M., Xu L. (2020). Phase Ib clinical trial of the anti-frizzled antibody vantictumab (OMP-18R5) plus paclitaxel in patients with locally advanced or metastatic HER2-negative breast cancer. Breast Cancer Res. Treat..

[250] Liao H., Li X., Zhao L., Wang Y., Wang X., Wu Y., Zhou X., Fu W., Liu L., Hu H.G. (2020). A PROTAC peptide induces durable β-catenin degradation and suppresses Wnt-dependent intestinal cancer. Cell Discov..

[251] Sharma A., Mir R., Galande S. (2021). Epigenetic Regulation of the Wnt/beta-Catenin Signaling Pathway in Cancer. Front. Genet..

[252] Nguyen N.M., Cho J. (2022). Hedgehog Pathway Inhibitors as Targeted Cancer Therapy and Strategies to Overcome Drug Resistance. Int. J. Mol. Sci..

[253] Li Y., Song Q., Day B.W. (2019). Phase I and phase II sonidegib and vismodegib clinical trials for the treatment of paediatric and adult MB patients: A systemic review and meta-analysis. Acta Neuropathol. Commun..

[254] Danial C., Sarin K.Y., Oro A.E., Chang A.L. (2016). An Investigator-Initiated Open-Label Trial of Sonidegib in Advanced Basal Cell Carcinoma Patients Resistant to Vismodegib. Clin. Cancer Res..

[255] Bruzzese A., Martino E.A., Labanca C., Mendicino F., Lucia E., Olivito V., Fimognari F., Neri A., Morabito F., Vigna E. (2023). Glasdegib for the treatment of acute myeloid leukemia. Expert. Opin. Pharmacother..

[256] Harada K., Ohashi R., Naito K., Kanki K. (2020). Hedgehog Signal Inhibitor GANT61 Inhibits the Malignant Behavior of Undifferentiated Hepatocellular Carcinoma Cells by Targeting Non-Canonical GLI Signaling. Int. J. Mol. Sci..

[257] Kim J., Lee J.J., Kim J., Gardner D., Beachy P.A. (2010). Arsenic antagonizes the Hedgehog pathway by preventing ciliary accumulation and reducing stability of the Gli2 transcriptional effector. Proc. Natl. Acad. Sci. USA.

[258] Liang G., Liu M., Wang Q., Shen Y., Mei H., Li D., Liu W. (2017). Itraconazole exerts its anti-melanoma effect by suppressing Hedgehog, Wnt, and PI3K/mTOR signaling pathways. Oncotarget.

[259] Becher O.J. (2019). HDAC inhibitors to the rescue in sonic hedgehog medulloblastoma. Neuro-Oncol..

[260] Pannuti A., Foreman K., Rizzo P., Osipo C., Golde T., Osborne B., Miele L. (2010). Targeting Notch to target cancer stem cells. Clin. Cancer Res..

[261] Wang J., Sullenger B.A., Rich J.N. (2012). Notch signaling in cancer stem cells. Adv. Exp. Med. Biol..

[262] Shih Ie M., Wang T.L. (2007). Notch signaling, γ-secretase inhibitors, and cancer therapy. Cancer Res..

[263] Massard C., Azaro A., Soria J.C., Lassen U., Le Tourneau C., Sarker D., Smith C., Ohnmacht U., Oakley G., Patel B.K.R. (2018). First-in-human study of LY3039478, an oral Notch signaling inhibitor in advanced or metastatic cancer. Ann. Oncol..

[264] Smith D.C., Eisenberg P.D., Manikhas G., Chugh R., Gubens M.A., Stagg R.J., Kapoun A.M., Xu L., Dupont J., Sikic B. (2014). A phase I dose escalation and expansion study of the anticancer stem cell agent demcizumab (anti-DLL4) in patients with previously treated solid tumors. Clin. Cancer Res..

[265] Jimeno A., Moore K.N., Gordon M., Chugh R., Diamond J.R., Aljumaily R., Mendelson D., Kapoun A.M., Xu L., Stagg R. (2019). A first-in-human phase 1a study of the bispecific anti-DLL4/anti-VEGF antibody navicixizumab (OMP-305B83) in patients with previously treated solid tumors. Investig. New Drugs.

[266] Smith D.C., Chugh R., Patnaik A., Papadopoulos K.P., Wang M., Kapoun A.M., Xu L., Dupont J., Stagg R.J., Tolcher A. (2019). A phase 1 dose escalation and expansion study of Tarextumab (OMP-59R5) in patients with solid tumors. Investig. New Drugs.

[267] Vigolo M., Urech C., Lamy S., Monticone G., Zabaleta J., Hossain F., Wyczechowska D., Del Valle L., O’Regan R.M., Miele L. (2023). The Efficacy of CB-103, a First-in-Class Transcriptional Notch Inhibitor, in Preclinical Models of Breast Cancer. Cancers.

[268] Hanna G.J., Stathis A., Lopez-Miranda E., Racca F., Quon D., Leyvraz S., Hess D., Keam B., Rodon J., Ahn M.J. (2023). A Phase I Study of the Pan-Notch Inhibitor CB-103 for Patients with Advanced Adenoid Cystic Carcinoma and Other Tumors. Cancer Res. Commun..

[269] Kim K.J., Kim J.W., Sung J.H., Suh K.J., Lee J.Y., Kim S.H., Lee J.O., Kim J.W., Kim Y.J., Kim J.H. (2020). PI3K-targeting strategy using alpelisib to enhance the antitumor effect of paclitaxel in human gastric cancer. Sci. Rep..

[270] Garrido-Castro A.C., Saura C., Barroso-Sousa R., Guo H., Ciruelos E., Bermejo B., Gavila J., Serra V., Prat A., Pare L. (2020). Phase 2 study of buparlisib (BKM120), a pan-class I PI3K inhibitor, in patients with metastatic triple-negative breast cancer. Breast Cancer Res..

[271] Yam C., Xu X., Davies M.A., Gimotty P.A., Morrissette J.J.D., Tetzlaff M.T., Wani K.M., Liu S., Deng W., Buckley M. (2018). A Multicenter Phase I Study Evaluating Dual PI3K and BRAF Inhibition with PX-866 and Vemurafenib in Patients with Advanced BRAF V600-Mutant Solid Tumors. Clin. Cancer Res..

[272] Laranjeira A.B.A., Hollingshead M.G., Nguyen D., Kinders R.J., Doroshow J.H., Yang S.X. (2023). DNA damage, demethylation and anticancer activity of DNA methyltransferase (DNMT) inhibitors. Sci. Rep..

[273] Hu C., Liu X., Zeng Y., Liu J., Wu F. (2021). DNA methyltransferase inhibitors combination therapy for the treatment of solid tumor: Mechanism and clinical application. Clin. Epigenetics.

[274] Billam M., Sobolewski M.D., Davidson N.E. (2010). Effects of a novel DNA methyltransferase inhibitor zebularine on human breast cancer cells. Breast Cancer Res. Treat..

[275] Chen R., Zhang M., Zhou Y., Guo W., Yi M., Zhang Z., Ding Y., Wang Y. (2020). The application of histone deacetylases inhibitors in glioblastoma. J. Exp. Clin. Cancer Res..

[276] Karagiannis D., Rampias T. (2021). HDAC Inhibitors: Dissecting Mechanisms of Action to Counter Tumor Heterogeneity. Cancers.

[277] Qi J., Shi Y. (2020). Selective Targeting of Different Bromodomains by Small Molecules. Cancer Cell.

[278] Wang Z.Q., Zhang Z.C., Wu Y.Y., Pi Y.N., Lou S.H., Liu T.B., Lou G., Yang C. (2023). Bromodomain and extraterminal (BET) proteins: Biological functions, diseases, and targeted therapy. Signal Transduct. Target. Ther..

[279] Wu D., Khan F.A., Zhang K., Pandupuspitasari N.S., Negara W., Guan K., Sun F., Huang C. (2024). Retinoic acid signaling in development and differentiation commitment and its regulatory topology. Chem. Biol. Interact..

[280] Brown G. (2023). Retinoic acid receptor regulation of decision-making for cell differentiation. Front. Cell Dev. Biol..

[281] Marcinkowska E., Wallace G.R., Brown G. (2016). The Use of 1alpha,25-Dihydroxyvitamin D(3) as an Anticancer Agent. Int. J. Mol. Sci..

[282] Duffy M.J., Murray A., Synnott N.C., O’Donovan N., Crown J. (2017). Vitamin D analogues: Potential use in cancer treatment. Crit. Rev. Oncol. Hematol..

[283] Schiera G., Di Liegro C.M., Di Liegro I. (2021). Involvement of Thyroid Hormones in Brain Development and Cancer. Cancers.

[284] Samudio I., Harmancey R., Fiegl M., Kantarjian H., Konopleva M., Korchin B., Kaluarachchi K., Bornmann W., Duvvuri S., Taegtmeyer H. (2010). Pharmacologic inhibition of fatty acid oxidation sensitizes human leukemia cells to apoptosis induction. J. Clin. Investig..

[285] De Los Santos-Jimenez J., Rosales T., Ko B., Campos-Sandoval J.A., Alonso F.J., Marquez J., DeBerardinis R.J., Mates J.M. (2023). Metabolic Adjustments following Glutaminase Inhibition by CB-839 in Glioblastoma Cell Lines. Cancers.

[286] Restall I.J., Cseh O., Richards L.M., Pugh T.J., Luchman H.A., Weiss S. (2020). Brain Tumor Stem Cell Dependence on Glutaminase Reveals a Metabolic Vulnerability through the Amino Acid Deprivation Response Pathway. Cancer Res..

[287] Cioce M., Pulito C., Strano S., Blandino G., Fazio V.M. (2020). Metformin: Metabolic Rewiring Faces Tumor Heterogeneity. Cells.

[288] Moufarrij S., Srivastava A., Gomez S., Hadley M., Palmer E., Austin P.T., Chisholm S., Diab N., Roche K., Yu A. (2020). Combining DNMT and HDAC6 inhibitors increases anti-tumor immune signaling and decreases tumor burden in ovarian cancer. Sci. Rep..

[289] Huang J.L., Chen S.Y., Lin C.S. (2022). Targeting Cancer Stem Cells through Epigenetic Modulation of Interferon Response. J. Pers. Med..

[290] Beziaud L., Young C.M., Alonso A.M., Norkin M., Minafra A.R., Huelsken J. (2023). IFNgamma-induced stem-like state of cancer cells as a driver of metastatic progression following immunotherapy. Cell Stem Cell.

[291] Lee K.W., Yam J.W.P., Mao X. (2023). Dendritic Cell Vaccines: A Shift from Conventional Approach to New Generations. Cells.

[292] Zhang D., Tang D.G., Rycaj K. (2018). Cancer stem cells: Regulation programs, immunological properties and immunotherapy. Semin. Cancer Biol..

[293] Cui P., Li R., Huang Z., Wu Z., Tao H., Zhang S., Hu Y. (2020). Comparative effectiveness of pembrolizumab vs. nivolumab in patients with recurrent or advanced NSCLC. Sci. Rep..

[294] Fessas P., Lee H., Ikemizu S., Janowitz T. (2017). A molecular and preclinical comparison of the PD-1-targeted T-cell checkpoint inhibitors nivolumab and pembrolizumab. Semin. Oncol..

[295] Chao M.P., Takimoto C.H., Feng D.D., McKenna K., Gip P., Liu J., Volkmer J.P., Weissman I.L., Majeti R. (2019). Therapeutic Targeting of the Macrophage Immune Checkpoint CD47 in Myeloid Malignancies. Front. Oncol..

[296] Masoumi J., Jafarzadeh A., Abdolalizadeh J., Khan H., Philippe J., Mirzaei H., Mirzaei H.R. (2021). Cancer stem cell-targeted chimeric antigen receptor (CAR)-T cell therapy: Challenges and prospects. Acta Pharm. Sin. B.

[297] Alhabbab R.Y. (2020). Targeting Cancer Stem Cells by Genetically Engineered Chimeric Antigen Receptor T Cells. Front. Genet..

[298] Wang W., Liu Y., He Z., Li L., Liu S., Jiang M., Zhao B., Deng M., Wang W., Mi X. (2024). Breakthrough of solid tumor treatment: CAR-NK immunotherapy. Cell Death Discov..

[299] Xiao L., Cen D., Gan H., Sun Y., Huang N., Xiong H., Jin Q., Su L., Liu X., Wang K. (2019). Adoptive Transfer of NKG2D CAR mRNA-Engineered Natural Killer Cells in Colorectal Cancer Patients. Mol. Ther..

[300] Benner B., Good L., Quiroga D., Schultz T.E., Kassem M., Carson W.E., Cherian M.A., Sardesai S., Wesolowski R. (2020). Pexidartinib, a Novel Small Molecule CSF-1R Inhibitor in Use for Tenosynovial Giant Cell Tumor: A Systematic Review of Pre-Clinical and Clinical Development. Drug Des. Dev. Ther..

[301] Rolfo C., Giovannetti E., Martinez P., McCue S., Naing A. (2023). Applications and clinical trial landscape using Toll-like receptor agonists to reduce the toll of cancer. NPJ Precis. Oncol..

[302] Kaczanowska S., Joseph A.M., Davila E. (2013). TLR agonists: Our best frenemy in cancer immunotherapy. J. Leukoc. Biol..

[303] Ciardiello D., Elez E., Tabernero J., Seoane J. (2020). Clinical development of therapies targeting TGFβ: Current knowledge and future perspectives. Ann. Oncol..

[304] Huang C.Y., Chung C.L., Hu T.H., Chen J.J., Liu P.F., Chen C.L. (2021). Recent progress in TGF-βinhibitors for cancer therapy. Biomed. Pharmacother..

[305] Fox E., Bates S.E. (2007). Tariquidar (XR9576): A P-glycoprotein drug efflux pump inhibitor. Expert. Rev. Anticancer Ther..

[306] Zechner M., Castro Jaramillo C.A., Zubler N.S., Taddio M.F., Mu L., Altmann K.H., Kramer S.D. (2023). In Vitro and In Vivo Evaluation of ABCG2 (BCRP) Inhibitors Derived from Ko143. J. Med. Chem..

[307] Packeiser E.M., Engels L., Nolte I., Goericke-Pesch S., Murua Escobar H. (2023). MDR1 Inhibition Reverses Doxorubicin-Resistance in Six Doxorubicin-Resistant Canine Prostate and Bladder Cancer Cell Lines. Int. J. Mol. Sci..

[308] Zhang Z., Patel S.B., King M.R. (2020). Micelle-in-Liposomes for Sustained Delivery of Anticancer Agents That Promote Potent TRAIL-Induced Cancer Cell Apoptosis. Molecules.

[309] Zhang Z., Xu X., Du J., Chen X., Xue Y., Zhang J., Yang X., Chen X., Xie J., Ju S. (2024). Redox-responsive polymer micelles co-encapsulating immune checkpoint inhibitors and chemotherapeutic agents for glioblastoma therapy. Nat. Commun..

[310] Blanpain C., Mohrin M., Sotiropoulou P.A., Passegue E. (2011). DNA-damage response in tissue-specific and cancer stem cells. Cell Stem Cell.

[311] Sperka T., Wang J., Rudolph K.L. (2012). DNA damage checkpoints in stem cells, ageing and cancer. Nat. Rev. Mol. Cell Biol..

[312] Shkundina I.S., Gall A.A., Dick A., Cocklin S., Mazin A.V. (2021). New RAD51 Inhibitors to Target Homologous Recombination in Human Cells. Genes.

[313] Paul S., Sinha S., Kundu C.N. (2022). Targeting cancer stem cells in the tumor microenvironment: An emerging role of PARP inhibitors. Pharmacol. Res..

[314] Wang Q.E. (2015). DNA damage responses in cancer stem cells: Implications for cancer therapeutic strategies. World J. Biol. Chem..

[315] Abad E., Graifer D., Lyakhovich A. (2020). DNA damage response and resistance of cancer stem cells. Cancer Lett..

[316] Galluzzi L. (2021). Targeting replication stress to tackle cancer stem cells. Cell Death Dis..

[317] Bhattacharjee S., Sullivan M.J., Wynn R.R., Demagall A., Hendrix A.S., Sindhwani P., Petros F.G., Nadiminty N. (2022). PARP inhibitors chemopotentiate and synergize with cisplatin to inhibit bladder cancer cell survival and tumor growth. BMC Cancer.

[318] Huang T.T., Lampert E.J., Coots C., Lee J.M. (2020). Targeting the PI3K pathway and DNA damage response as a therapeutic strategy in ovarian cancer. Cancer Treat. Rev..

[319] Cheng T., Rodrigues N., Shen H., Yang Y., Dombkowski D., Sykes M., Scadden D.T. (2000). Hematopoietic stem cell quiescence maintained by p21cip1/waf1. Science.

[320] Rampioni Vinciguerra G.L., Sonego M., Segatto I., Dall’Acqua A., Vecchione A., Baldassarre G., Belletti B. (2022). CDK4/6 Inhibitors in Combination Therapies: Better in Company Than Alone: A Mini Review. Front. Oncol..

[321] Kurppa K.J., Liu Y., To C., Zhang T., Fan M., Vajdi A., Knelson E.H., Xie Y., Lim K., Cejas P. (2020). Treatment-Induced Tumor Dormancy through YAP-Mediated Transcriptional Reprogramming of the Apoptotic Pathway. Cancer Cell.

[322] Mai Y., Su J., Yang C., Xia C., Fu L. (2023). The strategies to cure cancer patients by eradicating cancer stem-like cells. Mol. Cancer.

[323] Brown J.R., Chan D.K., Shank J.J., Griffith K.A., Fan H., Szulawski R., Yang K., Reynolds R.K., Johnston C., McLean K. (2020). Phase II clinical trial of metformin as a cancer stem cell-targeting agent in ovarian cancer. JCI Insight.

[324] Yap T.A., Daver N., Mahendra M., Zhang J., Kamiya-Matsuoka C., Meric-Bernstam F., Kantarjian H.M., Ravandi F., Collins M.E., Francesco M.E.D. (2023). Complex I inhibitor of oxidative phosphorylation in advanced solid tumors and acute myeloid leukemia: Phase I trials. Nat. Med..

[325] Liang D.H., Choi D.S., Ensor J.E., Kaipparettu B.A., Bass B.L., Chang J.C. (2016). The autophagy inhibitor chloroquine targets cancer stem cells in triple negative breast cancer by inducing mitochondrial damage and impairing DNA break repair. Cancer Lett..

[326] Marsh T., Tolani B., Debnath J. (2021). The pleiotropic functions of autophagy in metastasis. J. Cell Sci..

[327] Abbott A. (2025). Stem cells head to the clinic: Treatments for cancer, diabetes and Parkinson’s disease could soon be here. Nature.

[328] Zhang C.L., Huang T., Wu B.L., He W.X., Liu D. (2017). Stem cells in cancer therapy: Opportunities and challenges. Oncotarget.

[329] Lee J.Y., Hong S.H. (2020). Hematopoietic Stem Cells and Their Roles in Tissue Regeneration. Int. J. Stem Cells.

[330] Arwert E.N., Hoste E., Watt F.M. (2012). Epithelial stem cells, wound healing and cancer. Nat. Rev. Cancer.

[331] Horsley V. (2020). Skin in the Game: Stem Cells in Repair, Cancer, and Homeostasis. Cell.

[332] Tang X., Deng P., Li L., He Y., Wang J., Hao D., Yang H. (2024). Advances in genetically modified neural stem cell therapy for central nervous system injury and neurological diseases. Stem Cell Res. Ther..

[333] Wang X., Wang Y., Gou W., Lu Q., Peng J., Lu S. (2013). Role of mesenchymal stem cells in bone regeneration and fracture repair: A review. Int. Orthop..

[334] Li T.T., Wang Z.R., Yao W.Q., Linghu E.Q., Wang F.S., Shi L. (2022). Stem Cell Therapies for Chronic Liver Diseases: Progress and Challenges. Stem Cells Transl. Med..

[335] King A., Balaji S., Keswani S.G., Crombleholme T.M. (2014). The Role of Stem Cells in Wound Angiogenesis. Adv. Wound Care.

[336] Chu D.T., Nguyen T.T., Tien N.L.B., Tran D.K., Jeong J.H., Anh P.G., Thanh V.V., Truong D.T., Dinh T.C. (2020). Recent Progress of Stem Cell Therapy in Cancer Treatment: Molecular Mechanisms and Potential Applications. Cells.

[337] Stuckey D.W., Shah K. (2014). Stem cell-based therapies for cancer treatment: Separating hope from hype. Nat. Rev. Cancer.

[338] Enow J.A., Sheikh H.I., Rahman M.M. (2023). Tumor Tropism of DNA Viruses for Oncolytic Virotherapy. Viruses.

[339] Labusca L., Herea D.D., Mashayekhi K. (2018). Stem cells as delivery vehicles for regenerative medicine-challenges and perspectives. World J. Stem Cells.

[340] Joshi S., Allabun S., Ojo S., Alqahtani M.S., Shukla P.K., Abbas M., Wechtaisong C., Almohiy H.M. (2023). Enhanced Drug Delivery System Using Mesenchymal Stem Cells and Membrane-Coated Nanoparticles. Molecules.

[341] Minev T., Balbuena S., Gill J.M., Marincola F.M., Kesari S., Lin F. (2024). Mesenchymal stem cells—The secret agents of cancer immunotherapy: Promises, challenges, and surprising twists. Oncotarget.

